# Lgr5^+^ intestinal stem cells reside in an unlicensed G_1_ phase

**DOI:** 10.1083/jcb.201708023

**Published:** 2018-05-07

**Authors:** Thomas D. Carroll, Ian P. Newton, Yu Chen, J. Julian Blow, Inke Näthke

**Affiliations:** 1 Cell and Developmental Biology, University of Dundee, Dundee, Scotland, UK; 2 Centre for Gene Regulation and Expression, University of Dundee, Dundee, Scotland, UK

## Abstract

During late mitosis and the early G_1_ phase, the origins of replication are licensed by binding to double hexamers of MCM2–7. In this study, we investigated how licensing and proliferative commitment are coupled in the epithelium of the small intestine. We developed a method for identifying cells in intact tissue containing DNA-bound MCM2–7. Interphase cells above the transit-amplifying compartment had no DNA-bound MCM2–7, but still expressed the MCM2–7 protein, suggesting that licensing is inhibited immediately upon differentiation. Strikingly, we found most proliferative Lgr5^+^ stem cells are in an unlicensed state. This suggests that the elongated cell–cycle of intestinal stem cells is caused by an increased G_1_ length, characterized by dormant periods with unlicensed origins. Significantly, the unlicensed state is lost in *Apc*-mutant epithelium, which lacks a functional restriction point, causing licensing immediately upon G_1_ entry. We propose that the unlicensed G_1_ phase of intestinal stem cells creates a temporal window when proliferative fate decisions can be made.

## Abstract (precis)

Cell cycles of intestinal stem and transit-amplifying cells are poorly understood. Comparing total and DNA-bound Mcm2 in intact intestinal crypts, Carroll et al. show that most stem cells reside for long periods in the unlicensed G_1_ phase. In the unlicensed G_1_ phase, stem cells could interpret cues before resuming cell division.

## Introduction

Cell division is necessary for homeostasis of adult tissue. It allows for the replacement of aged or damaged cells and provides specialized cells critical for tissue function. The decision to proliferate is crucial, especially for stem cells, which produce daughter cells that either maintain the fate of a stem cell or differentiate to produce specialized cells. The rapidly renewing intestinal epithelium replenishes its cellular content every 4–5 d. This high turnover rate is maintained primarily by Lgr5^+^ intestinal stem cells in the crypt base, which are thought to be continually proliferative (Basak et al., 2014) as confirmed by proteomic and transcriptomic analysis (Muñoz et al., 2012). There is also a quiescent stem cell population that can reengage with the cell cycle to repopulate the Lgr5^+^ cell population if it becomes depleted. These quiescent stem cells reside at the +4 position and constitute a subset of Lgr5^+^ cells and are immature, secretory-lineage precursors (Buczacki et al., 2013). Lgr5^+^ stem cells can divide to form transit-amplifying (TA) cells, which undergo several rounds of cell division before differentiating and losing proliferative competency (Potten and Loeffler, 1990).

How proliferative-fate decisions are governed in stem and TA cells is not understood. Lineage-tracing studies suggest that in homeostatic intestinal tissue only five to seven intestinal stem cells are “active” of the 12–16 Lgr5^+^ cells present in the crypt base (Kozar et al., 2013; Baker et al., 2014). Interestingly, Lgr5^+^ cells have a significantly longer cell cycle than do TA cells (Schepers et al., 2011). The functional significance of the prolonged cell-cycle time on Lgr5^+^ stem cells is currently unknown, but it suggests an active regulation of cell-cycle progression and proliferative fate commitment.

Proliferative-fate decisions are typically visualized by detecting markers that are present in all cell-cycle phases, which only distinguishes proliferative from quiescent cells. Visualizing the incorporation of labeled nucleosides, such as BrdU or 5-ethynyl-2′-deoxyuridine (EdU), marks cells in the S phase. The limitation of these methods is that they cannot discriminate early proliferative-fate decisions made during the preceding mitosis or during the early stages of G_1_. DNA replication in the S phase depends on origin licensing, which involves the regulated loading of minichromosome maintenance (MCM) 2–7 complexes onto origins of DNA replication (Blow and Hodgson, 2002; Champeris Tsaniras et al., 2014). During the S phase, DNA-bound MCM2–7 hexamers are activated to form the catalytic core of the DNA helicase as part of the CMG (Cdc45, MCM2–7, GINS) complex (Moyer et al., 2006; Ilves et al., 2010; Makarova et al., 2012). Replication licensing is thought to occur from late mitosis and throughout the G_1_ phase until passage through the restriction point (Dimitrova et al., 2002; Namdar and Kearsey, 2006; Symeonidou et al., 2013; Håland et al., 2015). Correspondingly, insufficient origin licensing directly limits the ability to progress past the restriction point causing cell-cycle arrest (Shreeram et al., 2002; Liu et al., 2009; Alver et al., 2014). When functional, this licensing-checkpoint can delay the S phase if an insufficient amount of origins have been licensed.

When cells enter the G_0_ phase, MCM2–7 proteins are down-regulated and degraded, primarily via E2F-mediated transcriptional control of *MCM2–7*, *Cdc6*, and *Cdt1* (Leone et al., 1998; Williams et al., 1998; Ohtani et al., 1999). This prevents terminally differentiated cells from reentering the cell cycle. In mammalian cells, artificial induction of quiescence through contact inhibition leads to gradual down-regulation of Cdc6 and MCM2–7 over several days (Kingsbury et al., 2005). These features have led to the suggestion that quiescence can be defined by an unlicensed state (Blow and Hodgson, 2002). Equally, the licensing status can define a different restriction point that signals proliferative-fate commitment at the end of mitosis and in early G_1_, independent of the retinoblast protein (Rb)/E2F restriction point.

The dynamics of replication licensing in the intricate cellular hierarchy of a complex, rapidly renewing adult tissue is not understood. Therefore, we investigated the licensing system in the intestinal epithelium, aiming to understand dynamics of early cell-cycle commitment in stem and TA cells and during terminal differentiation.

## Results

### Mcm2 expression declines along the crypt–villus axis

Because of their abundance and their strong conservation and association with the core DNA replication process, the presence of MCM2–7 proteins is commonly used to establish proliferative capacity in tissues, similar to Ki67 or PCNA (Williams et al., 1998; Stoeber et al., 2001; Gonzalez et al., 2005; Juríková et al., 2016). Usually, terminally differentiated cells in mammalian tissues do not contain MCM2–7 (Todorov et al., 1998; Stoeber et al., 2001; Eward et al., 2004). To establish the overall MCM2–7 protein abundance along intestinal crypts, we first examined the expression of MCM2–7 proteins in the epithelium of the small intestines of adult murine by high-resolution immunofluorescence microscopy. We focused on Mcm2 as a surrogate for all the members of the MCM2–7 complex, based on their similar function and localization. However, we repeated a subset of the experiments using an antibody to Mcm4, which is less effective in detecting endogenous proteins. Nonetheless, in all cases, the results were identical.

**Figure 1.**
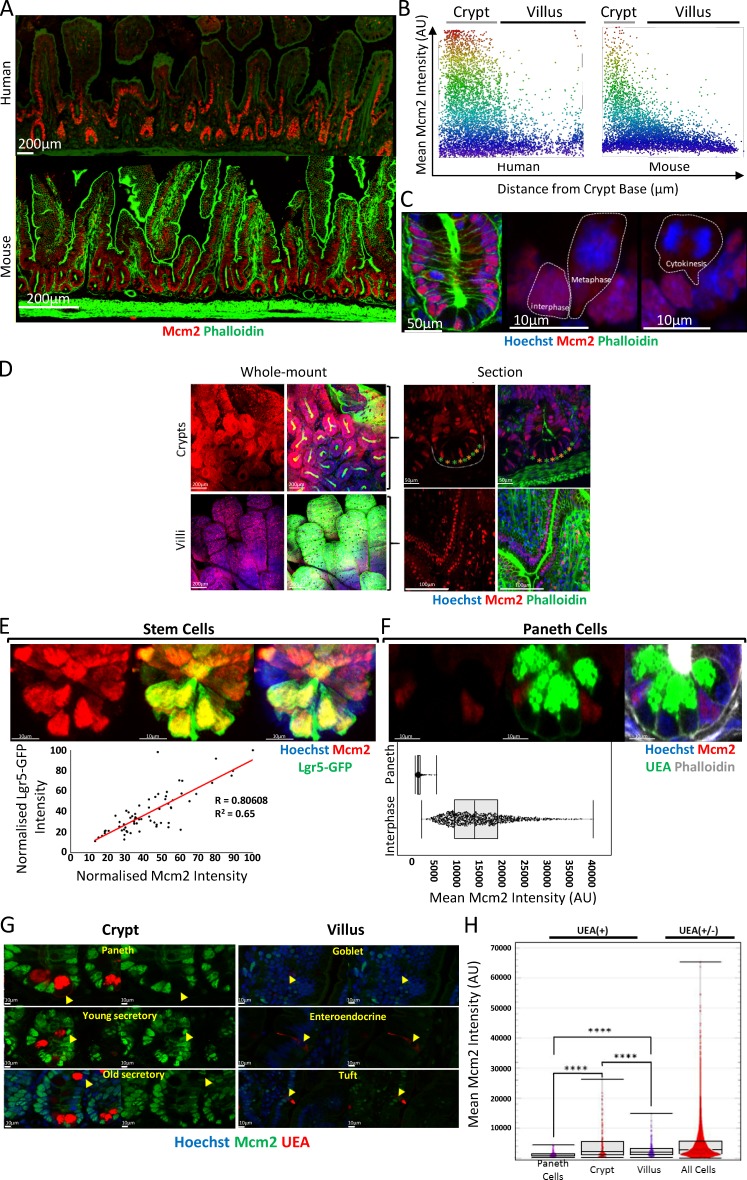
**Mcm2 is expressed ubiquitously along the crypt–villus axis and declines slowly as cells differentiate. (A)** Sections of normal human (top) and mouse (bottom) small intestine were stained with phalloidin (green) and an antibody against Mcm2 (red). Bars, 200 µm. **(B)** Mean Mcm2 intensities for segmented nuclei were plotted along the crypt–villus axis for human (left) and mouse (right) tissues. Locations of the crypt and villus domains are indicated. **(C)** An intestinal crypt stained with Hoechst (blue), phalloidin (green), and an antibody against Mcm2 (red). Individual cells in interphase and mitosis (metaphase and cytokinesis) are outlined by dashed white lines. Bars: (left) 50 µm; (middle and right) 10 µm. **(D)** Maximum-intensity projections of whole-mount intestinal tissue revealing intestinal crypts and villi (left; Bars, 200 µm). Individual X–Y sections are also shown to reveal the epithelium (right; Bars: [top] 50 µm; [bottom] 100 µm). Tissue was stained with phalloidin (green), Hoechst (blue), and an antibody against Mcm2 (red). The alternating pattern of Mcm2^+^ (green stars) and Mcm2^−^ (orange stars) in the crypt base is highlighted. **(E)** Images of Lgr5–GFP stem cells (green; top) costained with an Mcm2 antibody (red). Bars, 10 µm. The correlation (Pearson’s correlation *R* = 0.81, P < 0.0001) between mean Mcm2 and Lgr5–GFP intensities for Lgr5–GFP^+^ cells (*n* = 69), normalized to the maximum intensity for an individual crypt, is shown. **(F)** Images of UEA^+^ Paneth cells (top) costained with an Mcm2 antibody (red) and UEA (green). Bars, 10 µm. Mean Mcm2 intensity for segmented nuclei of UEA^+^ Paneth cells was compared with interphase cells (right). **(G)** Mcm2 (green) and UEA (red) expression in subsets of UEA^+^ cells in crypt and villus domains. Bars, 10 µm. UEA^+^ cells at the crypt base represent Paneth cells. **(H)** Quantification of mean Mcm2 intensity in individual UEA^+^ cell populations. UEA^+^ cells in the crypt base (Paneth cells, *n* = 224), in the upper crypt compartment (crypt, *n* = 132) and in the villus compartment (villus, *n* = 225) were identified manually, and the nuclear Mcm2 intensity was determined for individual cells (all cells, *n* = 33,736). There was a significant difference between UEA^+^ cells in the crypt and villus compartments (*t* test,****, P < 0.0001).

Consistent with previous studies, Mcm2 was highly expressed in both murine and human intestinal epithelium. Mcm2 was highly expressed in intestinal crypts (Fig. 1 A) and declined gradually along the crypt–villus axis (Fig. 1 B) but persisted in a few cells in the villus compartment (Fig. 1 D). Mcm2 was nuclear in interphase cells but cytoplasmic during mitosis (Fig. 1 C). Although most intestinal crypt cells expressed Mcm2, at the crypt base, Mcm2^+^ and Mcm2^−^ cells were interspersed (Fig. 1, A and D), consistent with previous studies (Pruitt et al., 2010). This pattern is reminiscent of the alternating arrangement of Lgr5^+^ stem cells and Paneth cells at the crypt base (Barker et al., 2007). Lgr5^+^ stem cells express Ki67 and are continually proliferative whereas Paneth cells are fully differentiated and are Ki67^−^ (Basak et al., 2014). As expected, Mcm2 was expressed in all Lgr5^+^ stem cells, and there was a strong correlation between Mcm2 and Lgr5 expression (Fig. 1 E). This is consistent with the idea that Lgr5^Hi^ stem cells are the main proliferative stem cells in the intestinal crypt. Staining with *Ulex europaeus* agglutinin (UEA) I demonstrated that most Mcm2^−^ cells in the crypt base are UEA^+^ Paneth cells (Fig. 1 F).

Normally, MCM2–7 expression is lost in terminally differentiated cells (Williams et al., 1998, 2004; Stoeber et al., 2001; Eward et al., 2004). The loss of expression has been suggested as a major contributor to the proliferation-differentiation switch in vivo*.* To test this idea, we measured the Mcm2 content of young and mature secretory cells in intestinal crypts and villi (Fig. 1, G and H). There was differential expression of Mcm2 in distinct secretory lineages. Many mature secretory cells, including Paneth, goblet, and enteroendocrine cells, were Mcm2^−^, consistent with their differentiation status and long life span in the epithelium (van der Flier and Clevers, 2009). We detected a few UEA^+^ Mcm2^+^ cells in the intestinal crypts (Fig. 1 G). If we assume that Mcm2 expression declines slowly after terminal differentiation, the presence of Mcm2 in UEA^+^ secretory cells could reflect their immaturity. Consistently, Mcm2 expression in UEA^+^ cells in the crypts was significantly greater than in the villi (Fig. 1 H), supporting the idea that MCM2–7 are gradually lost upon terminal differentiation. Because MCM2–7 are highly abundant and have a long (>24 h) half-life (Musahl et al., 1998), it likely that, after cells differentiate, their MCM2–7 content declines at a slow rate, explaining why Mcm2 persists in the villus compartment.

### Visualization of DNA replication licensing in vivo

**Figure 2.**
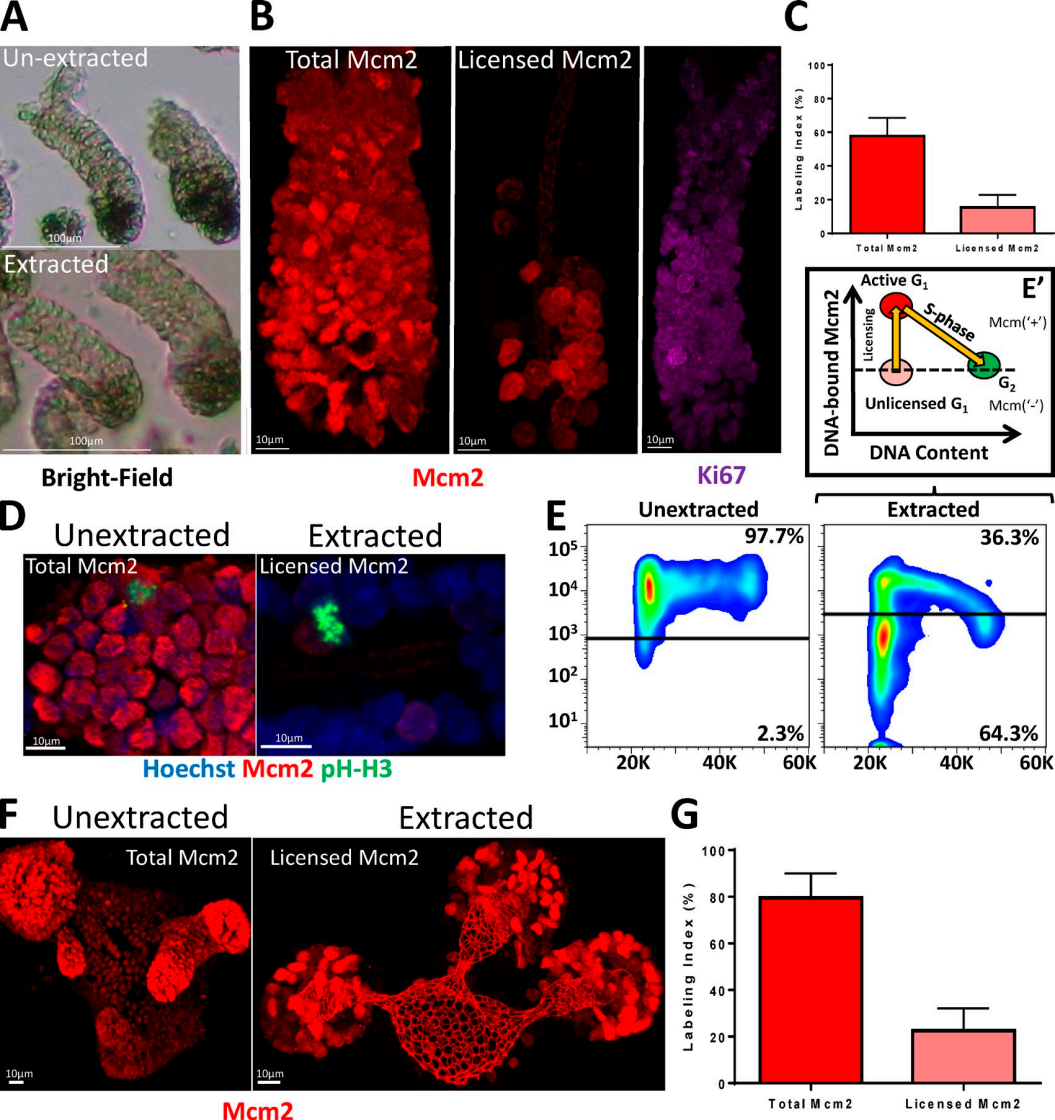
**Visualizing Mcm2 licensing in intestinal crypts. (A)** Representative bright-field images of extracted and unextracted, isolated intestinal crypts. Bars, 100 µm. **(B)** Representative images of isolated crypts stained with antibodies against Mcm2 (red) or Ki67 (purple). Bars, 10 µm. **(C)** The Mcm2 labeling index for unextracted and extracted crypts is significantly different (Means ± SEM, *n* = 10 crypts; *t* test, P < 0.0001). **(D)** Representative intestinal crypts stained with Hoechst (blue) and antibodies against Mcm2 (red) and phospho-histone H3 (pH-H3; green). Bars, 10 µm. **(E)** Representative flow cytometry profiles for extracted and unextracted, isolated, crypt epithelial cells showing Mcm2 versus DNA content. Data are representative of 3 independent experiments. **(E’)** Suggested model of the licensing profile shown in E. Deeply quiescent cells do not express Mcm2 and have a no detectable Mcm2 signal. Cells expressing soluble Mcm2 (unlicensed G_1_) show a similar Mcm2 signal to G_2_ cells. After a proliferative-fate decision has been made, origins become licensed and cells commit to S phase entry. Cells enter S phase after maximal origin licensing (active G_1_). During the S phase, Mcm proteins are then displaced from DNA during replication. **(F)** Representative images of extracted and unextracted intestinal organoids stained with an antibody against Mcm2 (red). Bars, 10 µm. **(G)** The Mcm2-labeling index for unextracted and extracted organoids. Data are displayed as means ± SEM; *n* = 3 organoids and shows a significant difference (*t* test, P < 0.0001).

MCM2–7 exist in three states: as hexamers, free in the nucleoplasm; as double hexamers, bound to DNA during late mitosis and G_1_ and S phases; or as CMG complexes, at replication forks during the S phase (Evrin et al., 2009; Remus et al., 2009; Gambus et al., 2011). To distinguish between DNA-bound and soluble forms, we developed a protocol involving a brief extraction of isolated crypts with nonionic detergent to remove soluble MCM2–7. The remaining Mcm2 should mark cells whose origins are licensed for replication. Extraction did not visibly affect intestinal crypt integrity but made them more opaque compared with unextracted tissue (Fig. 2 A). Most cells in unextracted crypts were Mcm2^+^ (Fig. 2 B, Total) similar to tissue sections and mirroring the ubiquitous expression of Ki67 along the crypt axis. After extraction, most of the Mcm2 content in cells was lost (Fig. 2 B, Licensed), with only 10–30% of cells maintaining high levels of Mcm2 (Fig. 2 C). After extraction, Mcm2^+^ was not present in mitotic cells expressing phosphorylated histone H3, confirming the extraction procedure successfully removed non–DNA-bound MCM2–7 proteins (Fig. 2 D).

We used flow cytometry to measure MCM2–7 content more directly and to further confirm the effectiveness of the extraction procedure. Whereas most isolated epithelial cells expressed Mcm2 that persisted throughout the cell cycle, extraction revealed a distinct profile of Mcm-containing cells in the crypts (Fig. 2 E). These profiles are consistent with those reported for cultured cell lines (Friedrich et al., 2005; Håland et al., 2015; Moreno et al., 2016; Matson et al., 2017). Mcm2 is present throughout the cell cycle (Fig. 2 E, Unextracted), but extraction shows that it binds to DNA throughout the G_1_ phase, reaching a maximum level before cells enter the S phase, and is subsequently displaced from the DNA during S phase. This behavior, which matches the known cell-cycle behavior of MCM2–7, confirms the efficiency of our extraction protocol. An antibody against Mcm4 produced similar results (data not shown). We observed that most cells with G_1_ DNA content appeared to be unlicensed, having a DNA-bound Mcm2 content similar to G_2_/M cells (Fig. 2, E and E′). This is substantially different from typical profiles observed in cultured cells lines in which most G_1_ cells are fully licensed (Friedrich et al., 2005; Håland et al., 2015; Moreno et al., 2016; Matson et al., 2017). Similar results were observed in cells isolated from intestinal organoids (Fig. 2, F and G).

### Licensing status and cell-cycle progression along the crypt–villus axis

Cell-cycle dynamics of intestinal stem and progenitor cells are highly heterogeneous (Pruitt et al., 2010). Most of the Lgr5^+^ stem cells are considered to be continually proliferative but with a much longer cell cycle than TA progenitor cells, which are most commonly found in the S phase (Schepers et al., 2011). To investigate proliferative-fate decisions of the intestinal epithelial cells, we used our MCM2–7 extraction in crypts in which S phase cells were labeled in vivo with the nucleoside analogue EdU. We then used image analysis software to correlate Mcm2 content with cell-cycle stage along the crypt–villus axis (Fig. S1, A–F). This allowed quantification of licensing in relation to the cell cycle and 3D spatial information.

**Figure 3.**
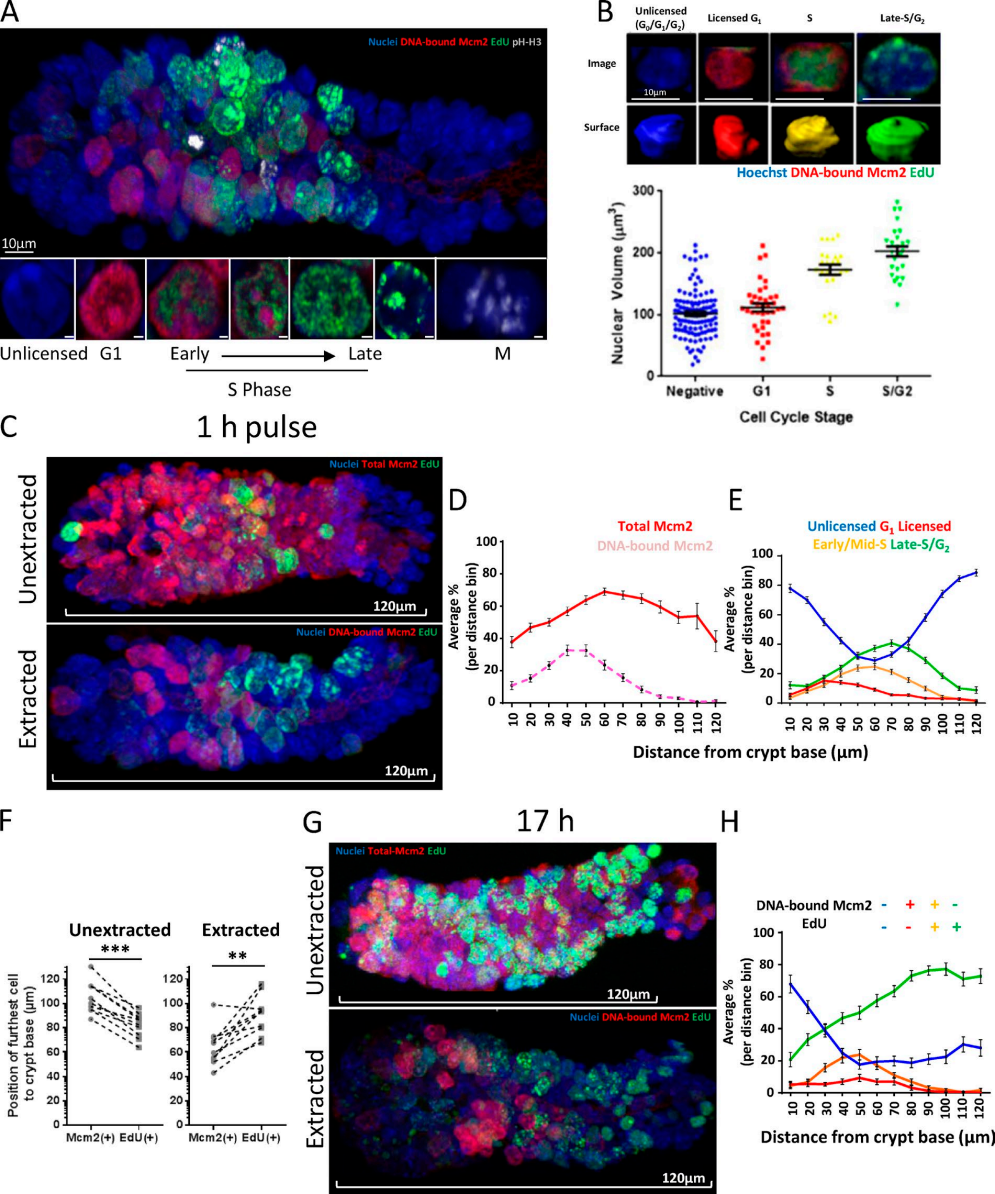
**The licensing state defines distinct proliferative zones in intestinal crypts. (A)** Representative image of an extracted intestinal crypt isolated after a 1-h EdU pulse in vivo (green) and stained with Hoechst (blue) and antibodies against Mcm2 (red) and pH H3 (white). Bars: (top) 10 µm; (bottom) 1 µm. Costaining shows distinct cell-cycle phases (bottom), licensed cells committed in G_1_ with Mcm2^+^ and EdU^−^; early Mcm2^+^ and EdU^+^ to late Mcm2^−^ and EdU^+^ in the S phase, and mitotic cells, pH H3^+^. Negative cells represent deeply quiescent (G_0_), terminally differentiated cells or cells in G_1_ that have not made a proliferative-fate decision, remaining unlicensed. The crypt base is to the left of the displayed image. **(B)** Nuclear volume was estimated in cells at the distinct cell-cycle phases identified previously: negative (G_0/_G_1_/G_2_, *n* = 115); G_1_ licensed Mcm^+^ and EdU^−^ (*n* = 38); S phase Mcm^+^ and EdU^+^ (*n* = 24); and late S/G_2_ Mcm^−^ and EdU^+^ (*n* = 26). (Top) Representative examples of each cell-cycle phase and the associated 3D rendered nuclei. Bars, 10 µm. The means ± SEM are displayed. There was a significant difference in the size of licensed G_1_, S, and late S/G_2_ nuclei (*t* test, P < 0.0001). **(C)** Representative images of intestinal crypts isolated after a 1-h EdU pulse (green) in vivo. Displayed are 3D projections of extracted and unextracted crypts stained with Hoechst (blue) and an antibody against Mcm2 (red). The crypt base is to the left of the displayed image. **(D)** Comparison between cells expressing Mcm2 protein and DNA-bound Mcm2 along the crypt–villus axis between unextracted (*n* = 101 crypts) and extracted (*n* = 109 crypts; taken from 3 mice) cells. Data are displayed as the mean percentage of cells per set distance bin. **(E)** All cells were divided into four distinct groups based on Mcm2 and EdU intensities. These groups represent distinctive cell-cycle phases as defined by their total (unextracted, *n* = 101 crypts) or licensed (extracted, *n* = 109 crypts) Mcm2 content: extracted, (1) Unlicensed Mcm2^−^ and EdU^−^, (2) G_1_ licensed Mcm2^+^ and EdU^−^, (3) early/mid S phase Mcm2^+^ and EdU^+^, and (4) late S/G_2_ Mcm2^−^ and EdU^+^. The data are represented as the population means of the total cells per distance bin. Means ± SEM; ***, P < 0.001; **, P < 0.01. **(F)** The distance of the most distal Mcm2^+^ and EdU^+^ cells to the crypt base was compared in extracted and unextracted crypts. Data were scored manually for 10 representative crypts per condition. Licensed Mcm2^+^ cells were significantly closer to the crypt base than were EdU^+^ cells (*t* test, P = 0.0015. Cells expressing Mcm2 protein extended significantly above the last EdU^+^ cell (*t* test, P < 0.0003). **(G)** Representative images of crypts isolated 17 h after administration of EdU (green). 3D projections of extracted and unextracted crypts stained with Hoechst (blue) and an antibody against Mcm2 (red) are shown. **(H)** Cells were divided into four distinct groups as in E (*n* = 51 crypts).

Fig. 3 A shows tissue labeled in vivo with a 1-h EdU pulse, followed by extraction of soluble MCM2–7. As expected, most cells in the TA compartment were labeled with EdU, suggesting that most cells were in the S phase, consistent with early studies with BrdU and [^3^H]thymidine labeling (Chwalinski and Potten, 1987). The patterns of replication foci were consistent with the reported S phase replication timing program (Rhind and Gilbert, 2013). Typically, all licensed cells had intense nuclear Mcm2 staining. Some cells completely lacked Mcm2 and EdU labeling, suggesting they were in either in G_0_, very early G_1_, or G_2_ phase. Some cells were labeled with both Mcm2 and EdU. These double-labeled cells typically showed patterns of EdU labeling consistent with early to mid S phase and Mcm2 labeling of DNA compartments expected to replicate later in S phase. This relationship has been observed in tissue-culture cells (Krude et al., 1996) and is consistent with the idea that DNA-bound MCM2–7 are displaced from chromosomal domains as replication is completed. Cells with late S phase patterns of EdU labeling had little or no detectable Mcm2, consistent with the displacement of most MCM2–7 by the end of the S phase. We also measured nuclear volume, which increases during S and G_2_ phases. This showed that nuclear volume increased up to twofold in cells classified as S and late S/G_2_ phases by Mcm2 and EdU staining (Fig. 3 B). This confirms our cell-cycle assignment and also suggests that most Mcm2^−^ cells are in G_0_ or G_1_, rather than in G_2_, phase.

The combination of concurrently labeling DNA-bound Mcm2 and EdU showed a clear correlation between cell position and cell-cycle stage. As noted previously, Mcm2 is expressed in cells throughout the crypt (Fig. 3, C and D). At the base of the crypt, unlicensed cells predominate (Fig. 3, C and E). At increasing distances from the crypt base, there is a successive rise in licensed G_1_, early/mid S, and then late S/G_2_ phase cells. Further up the crypt, at the end of the TA compartment, these cell-cycle stages decline in reverse order, until unlicensed cells again predominate. This suggests that there is a coordinated progression through the cell-division cycle as cells enter, then leave, the TA compartment. This was also observed as a field effect with many neighboring cells showing similar replication patterns (Fig. S2, A and B; and Video 1).

### Terminal differentiation is associated with a binary licensing decision

At the terminal boundary of the TA compartment, most cells were unlicensed and had no DNA-bound Mcm2 (Fig. 3, C and E). Similarly, there were no licensed G_1_ cells beyond the TA compartment, as defined by incorporation of EdU (Fig. 3 F). However, total Mcm2 expression extended significantly beyond the last cells with DNA-bound Mcm2 or incorporated EdU (Fig. 3, C, D, and F). The distribution of total Mcm2 expression corresponded to the zone in which cells express Ki67 (Fig. S3). Although Mcm2 and Ki67 expression persists beyond the TA compartment, licensing does not occur in this area. This suggests that differentiation is not governed by a gradual reduction in total MCM2–7 levels but is a binary decision, and licensing is abolished immediately after the final mitosis preceding differentiation. To further examine this, we marked the terminally differentiated zone by a 1-h EdU pulse, followed by a 16-h chase (Fig. 3, G and H). After 16 h, most of the distal end of the TA compartment became labeled with EdU. All labeled nuclei in this area were significantly smaller than EdU^+^ cells at the proximal end of the TA compartment (unpublished data), suggestive of their differentiation status. Importantly, the EdU^+^-differentiated cells at the distal end of the TA compartment lacked DNA-bound Mcm2, supporting our suggestion that licensing is inhibited immediately at terminal differentiation.

### Most intestinal stem cells spend most of their time in the G_1_ phase in an unlicensed state

**Figure 4.**
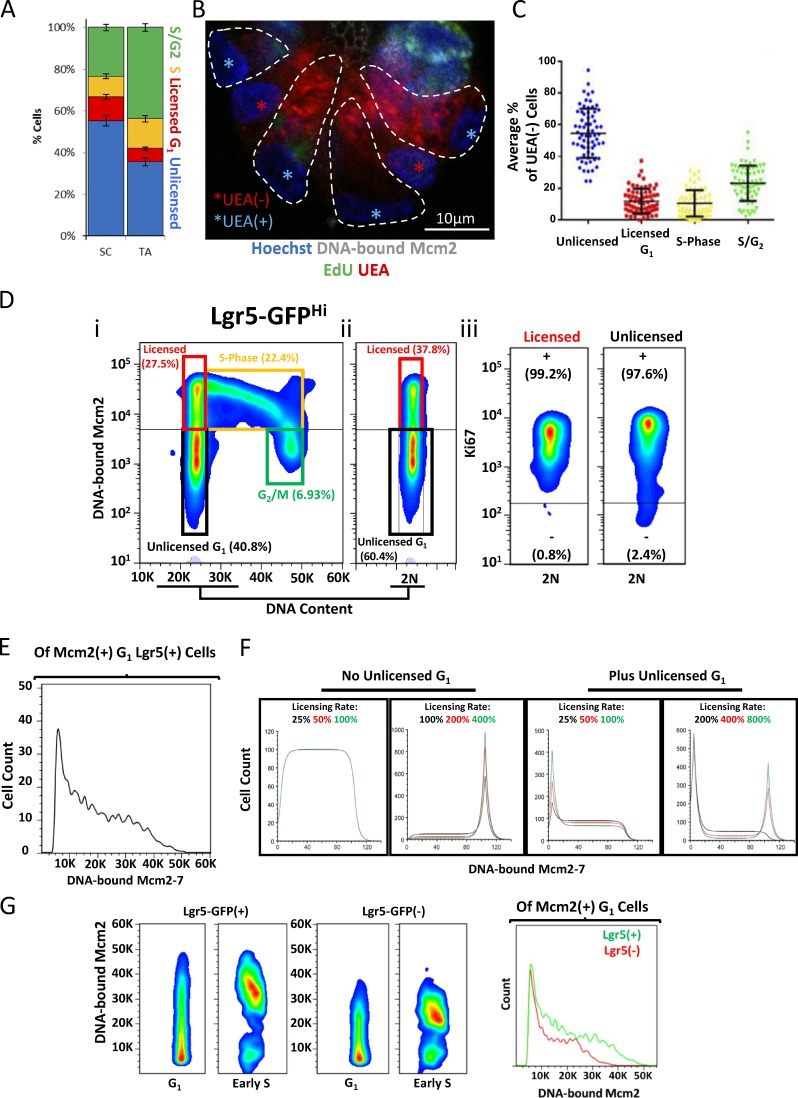
**Intestinal stem cells reside in a paused, unlicensed G_1_ phase. (A)** Quantification of cell-cycle stages for cells in the stem cell (SC) compartment (<40 µm from the crypt base) and in the early TA compartment (40–80 µm from the crypt base), as described in Fig. 3 E (*n* = 49 crypts). **(B)** Representative image of an extracted crypt base isolated 1 h after a pulse of EdU (green) and stained with Hoechst (blue), UEA (red), and antibodies against Mcm2 (white). Bar, 10 µm. Nuclear morphology and UEA signals were used to distinguish between UEA^+^ Paneth cells (outlined by dashed lines and nuclei marked with blue stars) and UEA^−^ stem cells (situated between outlined Paneth cells, with nuclei marked by red stars). **(C)** The mean percentage of UEA^−^ stem cells that fall into the previously defined cell-cycle bins: unlicensed; G_1_ licensed; S phase; and S/G_2_ phase (*n* = 68 crypts). Displayed are the means ± SEM. **(D)** Representative flow cytometry profiles of isolated Lgr5^Hi^ intestinal stem, showing DNA-bound Mcm2 and DNA content (*n* = 3 mice; panel i). The 2N (G_1_) cells of the same population are also shown (ii). The respective gated populations for unlicensed G_1_ (black), licensed (red), S phase (yellow), and G_2_/M (green) are shown. The Ki67 content of the licensed and unlicensed G_1_ cells are also shown (iii). **(E)** The frequency distribution of mean DNA-bound Mcm2 intensities for Mcm2^+^ cells in G_1_ cells. Shown are pooled data from three mice. **(F)** Simulated ergodic-rate analysis of origin licensing during G_1_, with the licensing rate varied and a significant paused period incorporated (unlicensed G_1_). The displayed histograms show the frequency distribution of DNA-bound Mcm2 of G_1_ cells (*n* = 10,000). Please refer to Materials and methods for further information. However, an analogy to explain the model is as follows, as described in Matson et al. (2017): Origin licensing through G_1_ can be thought of as cars traveling along a long stretch of motorway; if cars enter the motorway at a fixed rate (i.e., unsynchronized cell cycles, cells entering G_1_), the density of the cars at any one point is inversely proportional to the speed at which they are traveling (“speed” in this case means the rate of licensing). We have added the concept of delays at the start and end, like tollbooths. In our model, there is a minimum drive time (minimum length of G_1_ required for cells to grow to a critical size before they enter the S phase; arbitrarily set at 100%). If the speed of the cars means they don’t reach the end of the motorway before that time is up, cars maintain a constant speed along the entire road and exit the motorway as soon as they reach the end. If cars drive faster, and they reach the end before that time is up (>100%), they have to wait at the end of the motorway until the critical time has expired, resulting in a peak accumulation at the fully licensed point. Unlicensed G_1_ is an enforced time that cars have to wait once they enter the motorway before they are allowed to drive along it. This creates a peak on the left (unlicensed cells). If the cars then drive slowly, when they get to the end of the motorway, they exit immediately because the critical time has expired (center), but if they drive fast enough, they reach the end of the motorway before the critical time has expired. This results in a peak accumulation at both minimally and maximally licensed points. **(G)** Comparison of DNA-bound Mcm2 content of Lgr5^+^ and Lgr5^−^ G_1_ cells and of cells in the very early S phase. Shown are pooled data from three mice.

Most cells in the crypt base expressed Mcm2, consistent with the finding that all Lgr5^+^ cells express Mcm2, but mature secretory cells, such as Paneth cells, do not (Fig. 1, D and E). Surprisingly, extraction revealed that only 7–15% of cells were licensed in the crypt base (Fig. 3, C and D), with most cells in an unlicensed state despite expressing Mcm2. The abundance of licensed cells peaked 40–60 µm away from the crypt base, corresponding to just above the +4/+5 cell position (Fig. 3, D and E). This suggests that most stem cells remained unlicensed. In contrast, most TA cells appear to progress rapidly through the cell cycle, and many more actively incorporate EdU (Fig. 4 A).

We next confirmed that the unlicensed cells at the crypt base were Lgr5^+^ stem cells. Because it is not possible to identify Lgr5 in these experiments, because it is extracted along with unbound Mcm2, we instead identified Paneth cells by UEA staining and considered all UEA^−^ cells in the crypt base as stem cells (Fig. 4 B). More than 50% of the UEA^−^ stem cells were in an unlicensed state and were not incorporating EdU (Fig. 4 C). Approximately 30–40% of all UEA^−^ cells in the stem cell compartment were in an active phase of the cell cycle (licensed G_1_, S, or G_2_; Fig. 4 C), corresponding to five to six stem cells of the total of 14 present (Snippert et al., 2010). This number is similar to the small number of proposed “working” stem cells in the crypt base (Kozar et al., 2013; Baker et al., 2014). Unlicensed cells not incorporating EdU (i.e., unlabeled cells in this experiment) could theoretically be in either G_1_ or G_2_ phase. To distinguish between these possibilities, we first isolated crypt cells from Lgr5–GFP mice and measured both GFP and DNA content. Both Lgr5^+^ and Lgr5^−^ cell populations had a similar cell-cycle profile with most cells having two complete sets of chromosomes (2N) DNA content (Fig. S2 C). We also examined the nuclear volume of cells at different positions along the crypt axis after staining for EdU incorporation and DNA-bound Mcm2. Most unlicensed cells had a nuclear volume similar to that of fully licensed cells in G_1_ and not cells in late S/G_2_ phase (Fig. S2 D). Together, these results suggest that, although they express abundant Mcm2, most intestinal stem cells reside in an unlicensed G_1_ state.

To confirm this conclusion, we flow-sorted unextracted Lgr5–GFP^+^ cells, extracted unbound MCM2–7, and stained the cells for Mcm2 and Ki67. Consistent with our previous results, most Lgr5^+^ cells with a 2N DNA content had low levels of DNA-bound Mcm2 and were in an unlicensed state (Fig. 4 D [i and ii]). Importantly, both the licensed and unlicensed cells were Ki67^+^ indicating that they had not withdrawn from the cell-cycle long-term (Fig. 4 D [ii]).

This unlicensed G_1_ state—2N DNA content, high Mcm2 expression, but low levels of DNA-bound Mcm2—could be explained by two slightly different scenarios: (1) MCM2–7 are loaded onto DNA slowly in Lgr5^+^ cells, thereby extending G_1_ (Schepers et al., 2011; Dalton, 2015); or (2) Most Lgr5^+^ cells enter G_1_ and remain in an unlicensed state but do not load MCM2–7 until an active decision is made to commit to cell-cycle progression and activate the licensing system, at which time, MCM2–7 proteins are rapidly loaded. In option (1), in which licensing is slow, the presence of unlicensed cells simply reflects the increased time required to fully license origins, and different levels of Mcm2 loading should be equally distributed among G_1_ cells. In option (2), however, in which G_1_ licensing does not occur during an early stage, there should be a discrete peak of unlicensed cells with G_1_ DNA content, representing cells that have withdrawn from the cell cycle, and there would be fewer G_1_ cells and they would be loaded with different amounts of MCM2–7. To distinguish between these two possibilities, we used ergodic rate analysis (Kafri et al., 2013; Matson et al., 2017). When examining the frequency distribution of DNA-bound Mcm2 in Lgr5^+^ cells with 2N DNA content, we found a discrete peak of unlicensed cells (Fig. 4 E), consistent with the second model. To confirm these observations, we performed computer modeling (see the online supplemental material for source code) in which the licensing rate was varied in the presence or absence of an initial G_1_ period when licensing did not occur (Fig. 4 F). With no unlicensed G_1_ period and low licensing rates (Fig. 4 F, left), G_1_ cells were equally distributed across the different degrees of licensing. With faster licensing rates and no unlicensed G_1_ period, a distinct peak of fully licensed G_1_ cells appeared, similar to what has been observed in cell lines. Only when an unlicensed G_1_ period was introduced did a distinct peak of unlicensed G_1_ cells appear (Fig. 4 F, left). Most of these unlicensed Lgr5^+^ cells express abundant Mcm2 (Fig. 1 D), which suggests that their G_1_ is characterized by a long unlicensed period. This may explain why the cell-cycle length of intestinal stem cells is significantly longer than it is for TA cells (Schepers et al., 2011).

To further confirm that many intestinal stem cells exist in an unlicensed G_1_ state, we examined the expression of fluorescence ubiquitination cell-cycle indicator (FUCCI) reporters as an independent indicator of cell-cycle progression. We harvested intestinal tissue from Fucci2aR mice (Mort et al., 2014) and examined the expression of the G_1_-specific hCdt1(30/120) and S/G_2_/M-specific hGeminin(1/110) reporters (Fig. S4 A). As expected, many TA cells were hGeminin(1/110)^+^. We also noticed that terminally differentiated cells, such as Paneth cells in the stem cell compartment, and cells at the tips of villi expressed high levels of hCdt1(30/120) (Fig. S4 B) reflecting accumulation of the reporter in differentiated cells (Mort et al., 2014). However, we found that most Mcm2^+^ stem cells in the crypt base expressed very low levels of the hCdt1(30/120) reporter, and only a few cells expressed high levels (Fig. S4, C and D). In contrast, most TA cells were either hGeminin(1/110)^high^ or hCdt1(30/120)^low^, consistent with a short G_1_ phase. Together, this suggests that stem cells remain in a paused, unlicensed G_1_ state, where they do not rapidly accumulate hCdt1(30/120), unlike rapidly proliferating cells.

Embryonic stem cells have been reported to license more replication origins than neural stem/progenitor cells differentiated from them (Ge et al., 2015). To determine whether adult stem and non–stem cells in intestinal crypts exhibit such differences, we compared the amount of DNA-bound Mcm2 in G_1_/G_0_ and early S phase Lgr5^+^ cells with that of Lgr5^−^ cells (Moreno et al., 2016). Although most Lgr5^+^ cells were unlicensed, when they entered the S phase, they had approximately twice as much DNA-bound Mcm2 as Lgr5^−^ cells had (Fig. 4 G). This is consistent with the idea that adult intestinal stem cells license more origins than TA cells do and may represent a mechanism to protect genomic integrity.

### Intestinal label-retaining cells are in a deep G_0_ state

**Figure 5.**
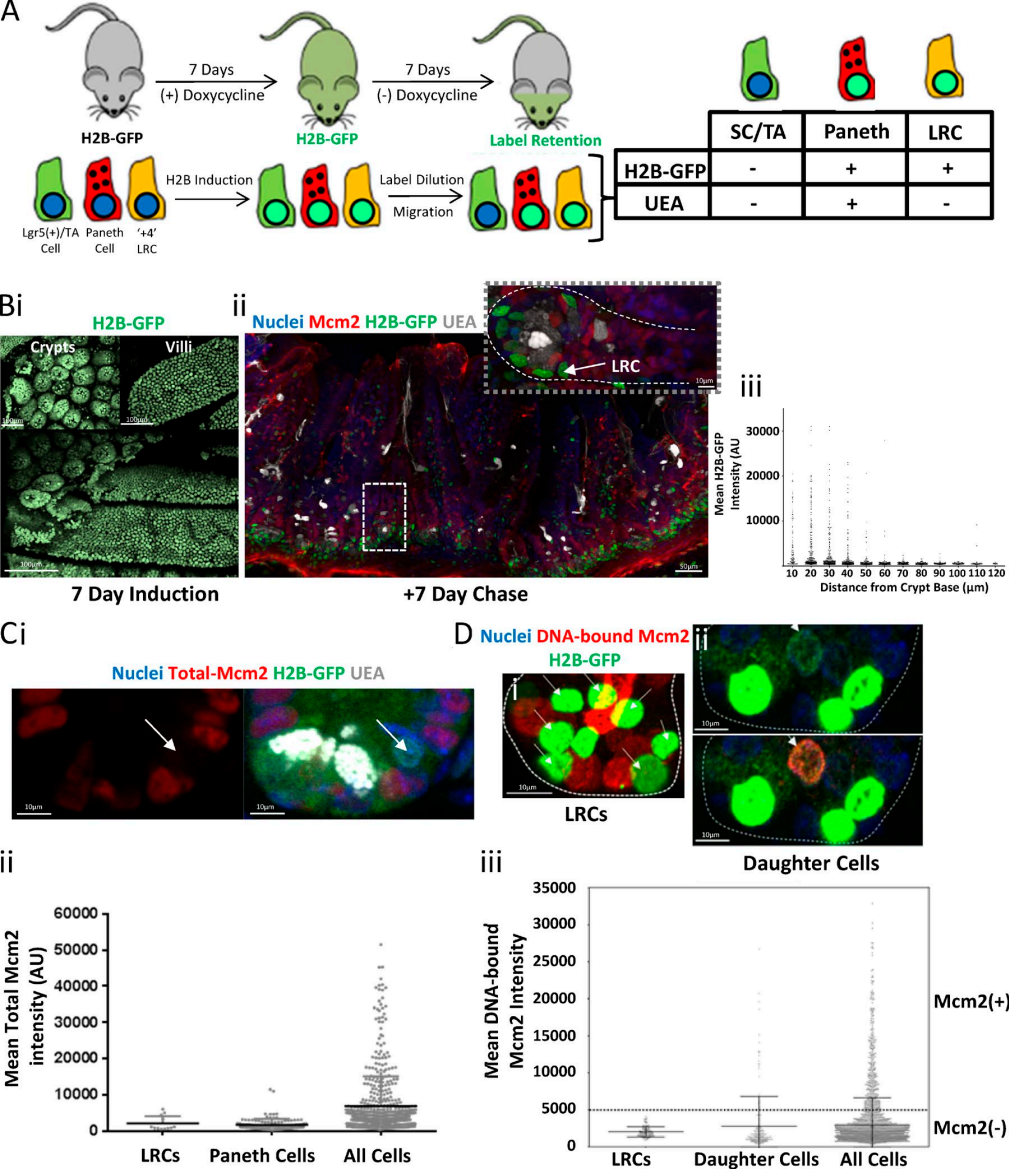
**LRCs are in a deep G_0_ state. (A)** Labeling strategy. H2B–GFP expression was induced in all intestinal epithelial cells in H2B–GFP mice by administration of doxycycline for 7 d. After complete labeling, doxycycline was removed, and mice rested for 7 d. During that chase period, most H2B–GFP^+^ cells are lost by label dilution because of cell division and upward migration. Both Paneth cells and +4 LRCs are H2B–GFP^+^ after the chase period. The +4 LRCs were distinguished from Paneth cells by the lack of UEA staining (Buczacki et al., 2013). **(B)** Representative images of whole-mount sections of H2B–GFP expressing small-intestine tissue after a 7-d labeling period (i). Bars, 100 µm. A vibratome section (ii) of intestinal tissue after a subsequent 7-d chase period, stained with Hoechst (nuclei), UEA (gray), and an antibody against Mcm2 (red). Bars, 50 µm. An enlarged image of the marked crypt is shown. Bar, 10 µm. A UEA^−^ LRC is marked with a white arrow. Quantification of the mean GFP intensity for all cells along the crypt–villus axis is shown after the 7-d chase period (*n* = 3,525 cells (iii). **(C)** A representative image (i) of an LRC in an intestinal crypt stained with Hoechst (blue), UEA (white), and an antibody against Mcm2 (red). Bar, 10 µm. The LRCs (white arrows) do not express Mcm2. The quantification of Mcm2 expression in LRCs (*n* = 12), Paneth cells (*n* = 116), and all cells (*n* = 543) is shown (iii). **(D)** Representative images of extracted H2B–GFP crypts after the 7-d chase period. Bar, 10 µm. H2B–GFP^Hi^ cells (bright green) represent Paneth cells and +4 LRCs (i), and H2B–GFP^low^ cells (faint green) represent daughter cells (white arrows) that have diluted H2B–GFP content because of cell division (ii). The quantification of DNA-bound Mcm2 in GFP^Hi^ LRCs (*n* = 110) and GFP^low^ daughter cells (*n* = 186) compared with the total cell population (all cells; *n* = 3,236) is shown (iii).

Although the intestinal crypt base primarily consists of Lgr5^+^ stem cells, there is also a reserved pool of quiescent stem cells, often referred to as “+4 label-retaining cells” (LRCs), reflecting their position in the crypt base and their ability to retain nascent DNA labels (Potten et al., 2002). These cells are a rare subset of Lgr5^+^ cells and are also secretory precursors (Buczacki et al., 2013). To further define the licensing status of these label-retaining, intestinal stem cells, we identified UEA^−^ LRCs by expressing H2B–GFP (which is incorporated into the chromatin of dividing cells) for 7 d and then chased them for a further 7 d (Roth et al., 2012; Buczacki et al., 2013). Labeled cells that did not divide during the 7-d chase period contained high levels of H2B–GFP (and were, therefore, LRCs), but cells that divided multiple times had only low levels of H2B–GFP. LRCs were then distinguished from Paneth cells based on UEA staining (Fig. 5 A). After induction, most cells in the epithelium expressed H2B–GFP (Fig. 5 B [i]). After the 7-d chase, H2B–GFP expression was restricted to cells near the villus tips, and cells at the crypt base (Fig. 5 B [ii]). We could successfully distinguish between Paneth cells and LRCs based on UEA staining (Fig. 5 B [ii]). Unlike most Lgr5^+^ cells, LRCs with high levels of GFP–H2B did not express Mcm2 (Fig. 5 C). As expected, only non-LRC daughter cells with low levels of H2B–GFP had DNA-bound Mcm2 (Fig. 5 D). This shows that LRC stem cells are in deep G_0_, unable to license because they do not express MCM2–7. In contrast, “active” intestinal stem cells mostly reside in an unlicensed G_1_ state.

### Licensing dynamics in intestinal organoids

**Figure 6.**
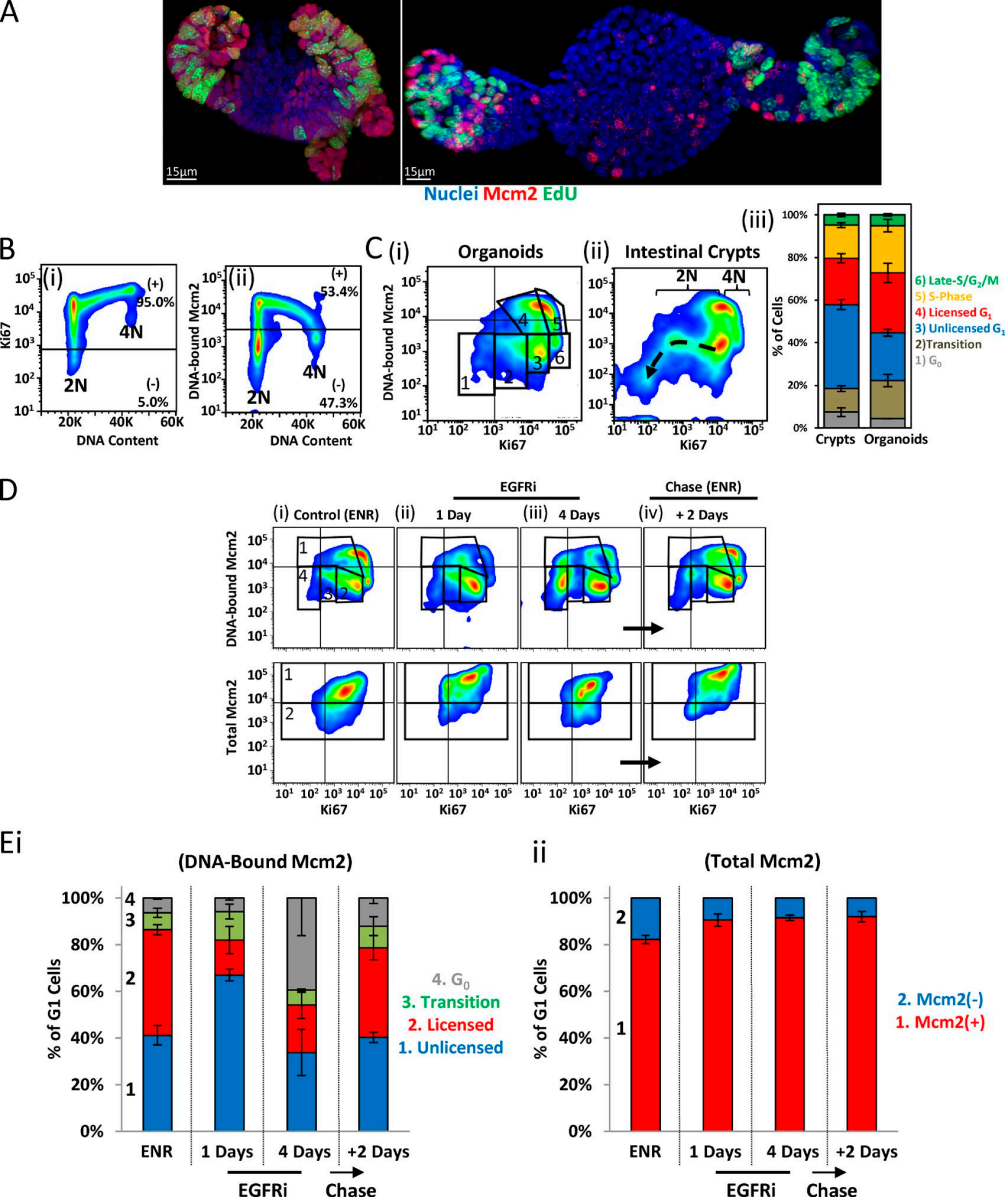
**Licensing dynamics in intestinal organoids. (A)** Representative image of unextracted and extracted intestinal organoids stained with Hoechst (blue) and an antibody against Mcm2 (red) after a 1-h EdU pulse (green). Bars, 15 µm. **(B)** Representative flow cytometry profiles from isolated organoid cells showing Ki67 (i) or DNA-bound Mcm2 (ii) content versus DNA content. Data are representative of three independent experiments. **(C)** Representative flow cytometry profile from cells isolated from cultured organoids (i) or intestinal crypts (ii) showing DNA-bound Mcm2 versus Ki67 (Fig. S5 A). Ki67 loss and subsequent loss of Mcm2 during differentiation is apparent, starting from unlicensed G_1_ (dashed arrow). Quantification of discrete populations (gates shown in i) was performed: (1) G_0_, (2) transition (G_0_ ↔ unlicensed G_1_), (3) unlicensed G_1_, and (4) licensed G_1_, (5) S phase, and (6) late S/G_2_/M (*n* = 3) with means ± SEM displayed. **(D)** Representative flow cytometry profiles of isolated organoid epithelial cells grown in ENR (control) and treated with the EGFR inhibitor gefitinib for the indicated times (i–iii). After 4 d in gefitinib, organoids were reactivated by removal of the gefitinib and readdition of fresh growth factors (ENR) for 2 d (iv). Displayed are profiles comparing DNA-bound Mcm2 versus Ki67 (top) or total Mcm2 content (bottom). Data are representative of three independent experiments. **(E)** The G_1_ cell populations, as shown in D, were quantified. DNA-bound Mcm2 profiles (i) show (1) unlicensed G_1_, (2) licensed G_1_, (3) transition (G_0_ ↔ unlicensed G_1_), and (4) G_0_ populations. Total-Mcm2 profiles (ii) show Mcm2-expressing, i.e., Mcm2^+^, and nonexpressing, i.e., Mcm2^−^, cells (*n* = 3). Data are displayed as percentage means ± SEM.

Intestinal stem cells reside in a highly specialized niche at the base of crypts. It is, therefore, possible that this niche specifically allows stem cells to pause in an unlicensed G_1_ state in which origin licensing is prevented until a further proliferative-fate signal is received. To understand the contribution of the stem cell niche to the dynamics of entry and exit from the unlicensed G_1_ state, we used intestinal organoids, which allowed us to manipulate the stem cell niche/environment with small molecules. Although the distribution of licensed cells was similar between the branches of intestinal organoids and crypts in tissue, there were considerably more cells with DNA-bound Mcm2 in the former (Fig. 6, A and C [iii]). Importantly, cells in organoids showed a discrete peak of fully licensed G_1_ cells in addition to the cells in the unlicensed G_1_ state (Fig. 6 B [ii]). This suggests that the epithelium in organoids represents an accelerated state of self-renewal and may not fully recapitulate cell-cycle dynamics of intestinal epithelial cells in vivo.

We designed an assay to robustly assess licensing dynamics during entry and exit from the unlicensed G_1_ state and the transition toward G_0._ Specifically, we used flow cytometry–based quantification of DNA-bound Mcm2, Ki67, and DNA content to measure licensing dynamics during entry and exit from the unlicensed G_1_ state and the transition toward G_0_ (Fig. S5 A). Most cells in organoids express Ki67, and it increased during cell-cycle progression (Fig. 6 B [i]). The DNA-bound Mcm2 profile was similar to that in isolated crypts (Fig. 6 B [ii]). Correlating Ki67 and DNA-bound Mcm2 produced a distinctive profile that is similar for isolated cells from organoids (Fig. 6 C [i]) and intestinal crypts (Fig. 6 C [ii]). This profile revealed a population of cells that appeared to lose Ki67 (Fig. 6 C [ii], dashed arrow) and may represent cells decreasing in proliferative capacity and transitioning toward differentiation (Fig. S5 A). Such a loss of proliferative capacity appeared to initiate in cells that expressed Ki67 but were unlicensed, i.e., cells in unlicensed G_1_ phase. These data suggest that different stages of quiescence can exist, which are reflected by a spectrum of Ki67 and Mcm levels.

Stem-cell niche maintenance in organoids mainly depends on a combination of EGF, Wnt, and Notch signaling (Sato et al., 2011). To identify the pathway that can modulate the unlicensed G_1_ state, we systematically treated organoids with a small molecule inhibitor of EGF receptor (EGFR; gefitinib), a Wnt agonist (Chir99021), and a Notch activator (valproic acid). Short-term treatment with gefitinib, which reduces mitogen-activated protein kinase activity and blocks DNA replication and cell division (Lynch et al., 2004), immediately caused cells to accumulate in the unlicensed G_1_ state with a 2N DNA content, but they continued to express Mcm2 and Ki67 (Fig. 6, D and E). Only prolonged EGFR inhibition (4 d) caused a transition to an intermediate G_0_ state, with significantly reduced Ki67 expression but with total Mcm2 levels maintained (Fig. 6, D and E). Both states were reversed by removal of EGFR inhibitors and addition of fresh EGFs (Fig. 6, D and E). Previously it was shown that EGFR increases Lgr5 expression (Basak et al., 2017), suggesting that these transitional states (unlicensed G_1_ and G_0_) are associated with “stemness.” Additionally, EGFR inhibitors appeared to potently kill TA cells (Fig. S5, B and C), leaving branches containing only stem cells (Basak et al., 2017). This suggests that both the fully quiescent G_0_ and the unlicensed G_1_ states can provide protection to stem cells.

Treatment with the Wnt agonist Chir99021 did not appear to significantly affect licensing dynamics (Fig. S5 C). Strikingly, treatment with valproic acid (a Notch activator) alone or in combination with Chir99021 significantly altered licensing profiles (Fig. S5, D and F). The combination of Chir99021 and valproic acid induced Lgr5 expression throughout the organoid epithelium (Fig. S5 E; Yin et al., 2014). Our data showed that this was associated with the appearance of a population of cells with low levels of Ki67 and intermediate levels of DNA-bound Mcm2 (Fig. S5 D), similar to the intermediate, unlicensed G_1_ state induced by EGFR inhibitors. Surprisingly, we observed an arc of cells connecting this to the fully licensed state, suggesting that relicensing of these cells occurs before they express high levels of Ki67 and that cells can reactivate licensing from a deeper state of G_0_ directly (Fig. S5, D and F).

Inhibiting Notch signaling with DAPT (*N*-[*N*-(3,5-difluorophenacetyl)-l-alanyl]-*S*-phenylglycine *t*-butyl ester), which induces terminal secretory cell differentiation (van Es et al., 2005), also significantly altered licensing dynamics and induced deep G_0_ with reduced Ki67 and loss of Mcm2 proteins (Fig. S5 G). Together, these data suggest that both EGFR and Notch signaling can significantly influence the licensing dynamics during the transition between quiescence and unlicensed G_1_.

### The unlicensed-G_1_ state is lost in *Apc* mutant organoids

Many established cell lines appear to lack an unlicensed G_1_ state; instead, licensing of all origins occurs immediately upon mitotic exit (Friedrich et al., 2005; Håland et al., 2015; Moreno et al., 2016; Matson et al., 2017). In addition, many of these cell lines lack a functional licensing checkpoint (Shreeram et al., 2002; Feng et al., 2003; Liu et al., 2009; Nevis et al., 2009), which has been suggested to arrest cells in G_1_ by inactivation of the RB-E2F restriction point (Shreeram et al., 2002; Machida et al., 2005; Teer et al., 2006; Liu et al., 2009; Nevis et al., 2009). To understand the biological relevance of the unlicensed G_1_ state, we determined whether origin licensing dynamics during G_1_ were altered during the initial stages of tumorigenesis.

**Figure 7.**
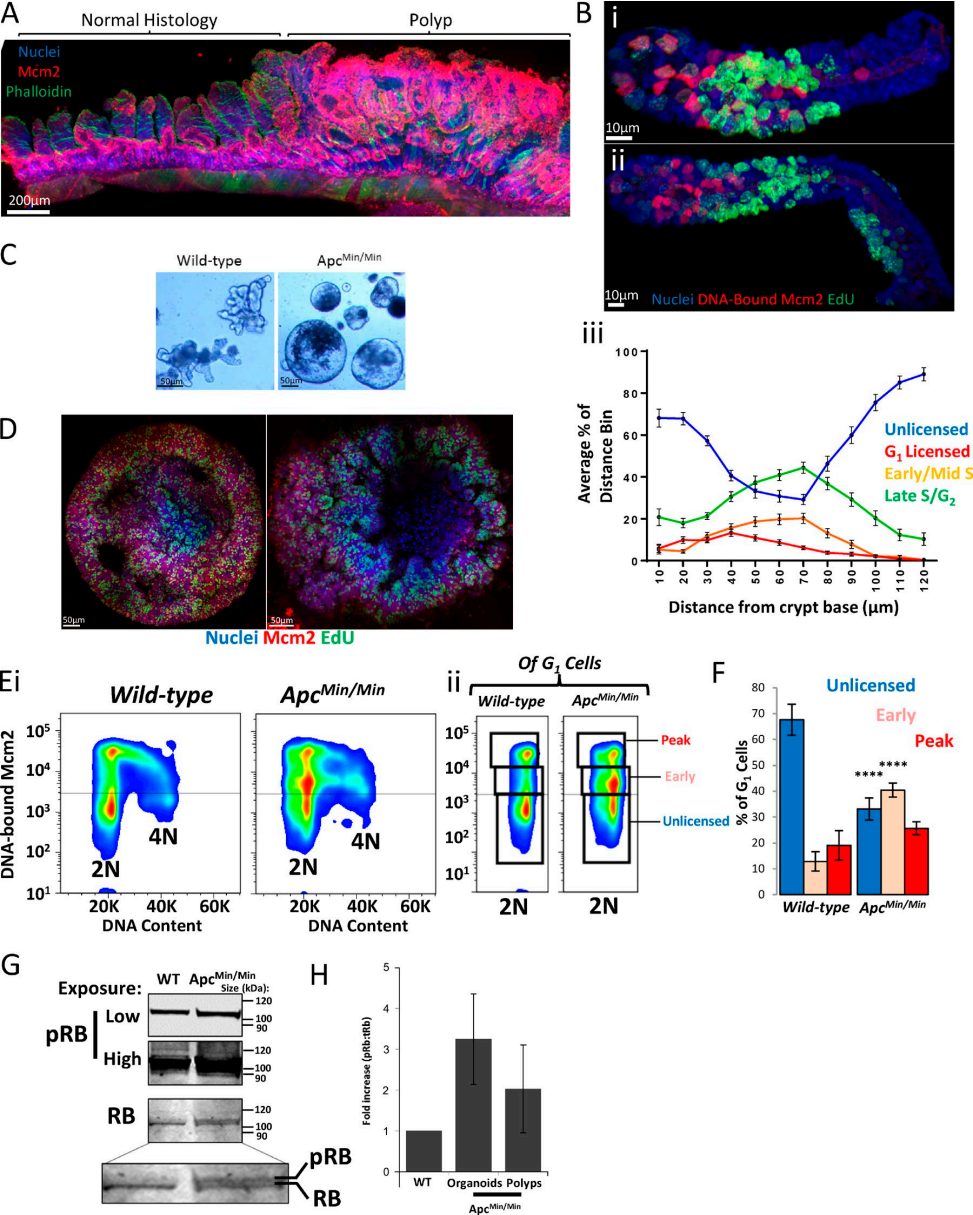
**Unlicensed G_1_ is lost in *Apc* mutant epithelium. (A)** A representative vibratome section of *Apc*^Min/+^ intestinal epithelium stained with Hoechst (nuclei), phalloidin, and an antibody against Mcm2. Bar, 200 µm. Regions of normal histology and a region containing a polyp are highlighted. **(B)** Representative image of an extracted *Apc*^Min/+^ isolated crypt (i) stained with Hoechst (nuclei) and an antibody against Mcm2 (red) after a 1-h EdU pulse (green). A representative image of an abnormally elongated crypt is displayed (ii) showing a clonal ribbon of cells in the distal TA compartment, which are EdU^+^. Quantification of cell-cycle stages across the crypt axis (Fig. 3) is shown (iii; *n* = 40 crypts). Bars, 10 µm. **(C)** Representative bright-field images of organoids cultured from WT and Apc^Min/+^ mice that have undergone LOH (Apc^Min/Min^). Bars, 50 µm. **(D)** Representative images of unextracted and extracted *Apc*^Min/Min^ organoids stained with Hoechst (nuclei) and an antibody against Mcm2 (red) after a 1-h EdU pulse. Bars, 50 µm. **(E)** Representative flow cytometry profiles from cells of extracted WT and *Apc*^Min/Min^ organoids showing DNA-bound Mcm2 versus DNA content (i). The 2N G_1_ cells of the profile shown are displayed (ii), showing unlicensed G_1_, early G_1_, and peak (fully licensed) G_1_ populations. Data are representative of three independent experiments. **(F)** Quantification of the populations described in E(ii). Data are displayed as means ± SEM (*n* = 3 organoids. **(G)** Western blot of Rb and pRb (low and high exposure) levels between WT and *Apc*^Min/Min^ organoids. Two bands, corresponding to hypo- and hyperphosphorylated Rb, are shown. **(H)** Quantification of the fold increase of pRb:Rb for Apc^Min/Min^ organoids (*n* = 3) and polyps isolated from Apc^Min/+^ mice (*n* = 6), compared with WT organoids and tissue.

The first initiating mutations in colorectal cancer are usually in *adenomatous polyposis coli* (*Apc*). Therefore, we investigated whether licensing dynamics were altered in *Apc-*mutant intestinal epithelium. In Apc^Min/+^ epithelium in vivo, Mcm2 expression appeared normal in areas of normal histology but was greatly increased in polyps (Fig. 7 A). In isolated Apc^Min/+^ crypts, there was a slight increase in the size of the proliferative compartment similar to that found in a previous study (Trani et al., 2014), and a slight increase in the number of EdU^+^ cells in the stem cell compartment (Fig. 7 B [i and iii]). However, in most crypts, there were no significant differences in the number or distribution of licensed cells in the stem-cell compartment (Fig. 7 B [i and iii]). However, we found a small number of crypts that were considerably larger. Strikingly, within these crypts, we noticed small “ribbons” of EdU^+^ cells that extended significantly into the TA compartment (Fig. 7 B [ii]). We suspected that these ribbons represent clones of cells that had undergone loss of heterozygosity (LOH) and that continually reengage with the cell cycle. This, in turn, suggests that the LOH event that converts *Apc^Min/+^* to *Apc^Min/Min^* cells significantly alters cell-cycle dynamics.

To determine whether LOH alters origin licensing dynamics by modifying G_1_, we compared WT and *Apc^Min/Min^* organoids (Fig. 7 C). *Apc^Min/Min^* organoids contained many more licensed cells that were distributed randomly throughout the organoid (Fig. 7 D), reminiscent of the altered distribution of Ki67^+^ cells in these organoids (Fatehullah et al., 2013). Strikingly, we found that licensing dynamics in the G_1_ phase of *Apc^Min/Min^* cells were different, and there was a significant loss of the unlicensed G_1_ population (Fig. 7 E). Instead, most cells appear to license immediately upon G_1_ entry and progressed into S phase immediately after minimal licensing (Fig. 7, E and F). Nonetheless, we still detected a population of cells that licensed as many origins as cells in WT organoids. These may be residual *Apc^Min/+^* cells that had not undergone LOH yet and thus maintained near-normal cell-cycle dynamics.

A functional licensing checkpoint depends on an intact Rb restriction point, and we tested whether the relative amounts of hypo- and hyperphosphorylated Rb varied between WT and *Apc^Min/Min^* epithelia. In WT organoids, we noticed that most of the Rb appeared hypophosphorylated (Fig. 7 G). In contrast, in *Apc^Min/Min^* organoids, at least half of the Rb appeared hyperphosphorylated, with at least threefold more phosphorylated Rb. Together, these data are consistent with the idea that cells exist in an unlicensed G_1_ state before passage through the restriction point and activation of E2F-driven transcription. In contrast, *Apc* mutant cells appear to have lost normal restriction-point control so that they have constitutively hyperphosphorylated Rb, allowing them to completely bypass the unlicensed G_1_ state.

## Discussion

The cell cycle of intestinal stem and TA cells is poorly understood. By comparing the total and DNA-bound Mcm2 in intact intestinal crypts, we provide new insights into how licensing and cell-cycle commitment are coupled in this tissue. We provide evidence that, after their final mitosis, TA cells do not license their replication origins and immediately exit the cell cycle. We show that many Lgr5^+^ stem cells spend most of the G_1_ phase in an unlicensed state, continually expressing Mcm2 and Ki67. In the unlicensed G_1_ phase, stem cells could be poised to respond to cues and progress past that restriction point to resume the cell division cycle.

**Figure 8.**
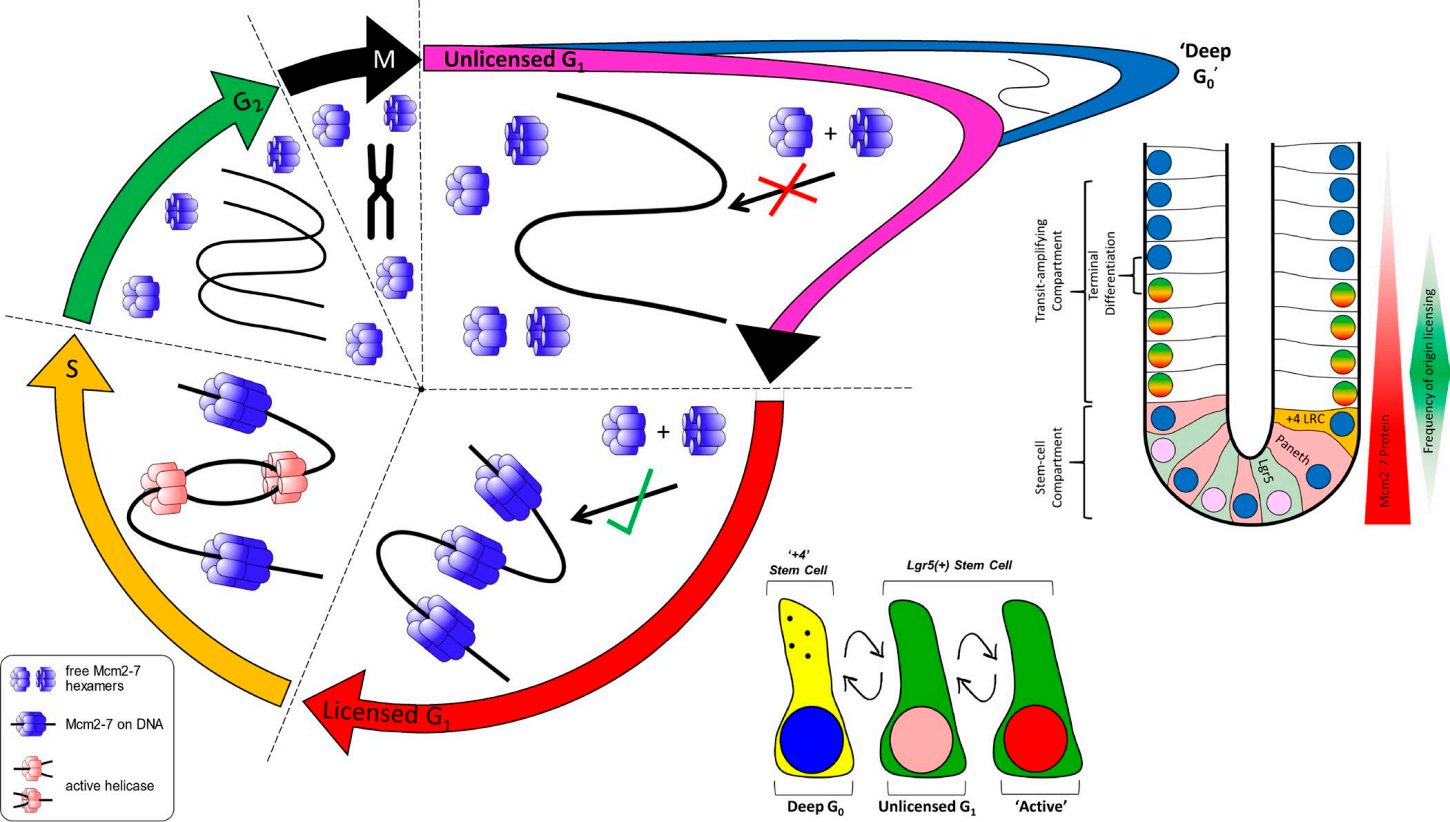
**Model of origin licensing dynamics in intestinal epithelial cells.** In a normal cell cycle, the Mcm cells are expressed ubiquitously in all stages. The licensing of DNA with MCM2–7 occurs in late M and throughout G_1,_ when a cell receives a stimulus to commit to the cell cycle. As DNA is replicated during the S phase, MCM2–7 are displaced from DNA and are prevented from relicensing in G_2_. During terminal differentiation, MCM2–7 are not actively transcribed, and the proteins are gradually lost in postmitotic cells. However, after the final mitotic division, cells make a binary decision never to license their DNA, even though the protein is still present. Mcm proteins then degrade slowly, where cells enter a terminally differentiated state (deep G_0_). Alternatively, cells can exit mitosis, not relicense their DNA but maintain proliferative markers, and disengage from the cell cycle for some time (unlicensed G_1_). Two major classes of intestinal stem cells exist: “active” stem cells, engaged with the cell cycle, and reserve, quiescent LRCs. LRCs are in a state of “deep” quiescence and do not contain MCM2–7 because they have disengaged from the cell cycle for some time. In this study, we show that most active Lgr5^+^ stem cells reside in an unlicensed state but contain MCM2–7 proteins. These cells reside in an unlicensed G_1_ phase until they make a proliferative-fate decision, enter the cell cycle, and license. This provides an explanation for the elongated cell cycle of intestinal stem cells; they reside in a partial resting state in which they may be able to respond to niche cues to divide. This, therefore, may constitute a unique mechanism to control stem cell numbers.

Lgr5^+^ stem cells have a cell-cycle length greater than TA cells (Schepers et al., 2011). The biological relevance of this is currently unknown. The data presented here suggest a delay in origin licensing is a key feature of the prolonged cell cycle of Lgr5^+^ cells. Although ∼80% of Lgr5^+^ cells are thought to be continually proliferative and express high levels of both Ki67 (Basak et al., 2014) and Mcm2, we found that most Lgr5^+^ cells reside in an unlicensed state, with 2N DNA content and Mcm2 not bound to DNA. Because the licensed state defines proliferative-fate commitment (Blow and Hodgson, 2002), we suggest that these cells are temporarily paused in the G_1_ phase, continuing to express proliferative makers, such as Ki67 and Mcm2, but without fully committing to the cell cycle (Fig. 8). We show that the number of Lgr5^+^ cells with DNA-bound Mcm2 was similar to the number of proposed active stem cells determined in lineage-tracing experiments (Kozar et al., 2013; Baker et al., 2014).

Prolonged arrest may eventually result in degradation of MCM2–7 proteins and lead to induction of a state of deep quiescence (G_0_). Consistent with this idea, we observed that LRCs, thought to provide a reserve of quiescent stem cells, did not express Mcm2. The lack of Mcm2 expression may reflect that significant time has passed since those cells divided. The delay in activating the licensing system may create a prolonged time window for Lgr5^+^ cells to receive and interpret environmental cues before deciding to commit to duplication, offering a means to control their number. It is likely that most Lgr5^+^ cells regularly resume their cell cycle, given their continual expression of proliferation markers (Basak et al., 2014). The identity and decisions of Lgr5^+^ cells are governed by stochastic choices, and the ability to pause briefly in G_1_ offers them unique flexibility in making those choices.

As expected, the transition from unlicensed G_1_ to licensed G_1_ seems dependent on EGFR signaling. However, other pathways responsible for stem-cell maintenance can also significantly cause the appearance of a unique population of unlicensed cells with distinct cell-cycle dynamics. Growing evidence suggests that intestinal stem-cell fate is not governed by asymmetric segregation of fate determinants (Lopez-Garcia et al., 2010; Snippert et al., 2010; Steinhauser et al., 2012). Instead, factors operating in the stem-cell niche, such as Wnt and Notch signaling, affect stem-cell fate decisions and also reduce the cycle rate of intestinal stem cells (Hirata et al., 2013). This is consistent with the idea that cell-fate choices are affected by decreasing proliferation rates and increasing G_0_/G_1_ length. Indeed, extending G_1_ in mouse and human embryonic stem cells can drive differentiation (Calder et al., 2013; Coronado et al., 2013). Similarly, long G_1_ phases are associated with the generation of fate-restricted progenitors during neurogenesis (Arai et al., 2011). An extended time window in the cell cycle has been suggested to allow niche factors and/or fate determinants to accumulate to direct progenitor fate (Calegari and Huttner, 2003). In the case of adult intestinal stem cells, holding cells in G_1_ may allow an extended time for stem-cell fate factors to act and maintain stem-cell fate. In contrast, many embryonic stem cells license rapidly, and the cell cycle slows throughout differentiation (Matson et al., 2017).

However, similar to embryonic stem cells (Ge et al., 2015), intestinal stem cells appear to have licensed more origins than non–stem cells have when they enter the S phase. This may help ensure accurate and complete genome duplication in long-lived stem cells (Moreno et al., 2016). With a greater demand for licensed origins, intestinal stem cells may, therefore, more readily engage the licensing checkpoint that ensures that all origins are licensed before cells enter the S phase (Shreeram et al., 2002; Liu et al., 2009; Alver et al., 2014). This additional demand for licensed origins in stem cells may also explain why crypts that are hypomorphic for Mcm2 have stem-cell deficiencies (Pruitt et al., 2007).

It is unclear how intestinal stem cells enter a significant, unlicensed G_1_ state. The simplest explanation is that licensing factors, such as Cdt1 or Cdc6, are not readily available in new-born stem cells, and their synthesis has to be stimulated by an upstream signal for fate commitment via activation of E2F-driven transcription. This is the situation after prolonged quiescence, which is accompanied by passive down-regulation of licensing factors (Coller, 2007). In contrast, in continually dividing cells, their levels are maintained. Consistent with this idea, licensing factors such as Cdc6, along with many cyclin-dependent kinase complexes, are down-regulated beyond the end of the TA zone (Frey et al., 2000; Smartt et al., 2007). Cells without a functional restriction point, such as *Apc* mutant cells or most cancer cell lines, could immediately license their origins upon entering G_1_ and can progress into S phase without sufficient origins being licensed. Interestingly, both TA cells and highly proliferative *Apc* mutant cells are very sensitive to replication inhibitors, such as gefitinib (Fig. S5 H). WT stem cells survive this treatment, potentially by engaging the licensing checkpoint to reversibly stall in the unlicensed G_1_ phase. This suggests that the unlicensed G_1_ phase can protect stem cells from replication inhibitors and offers researchers a potentially selective means to kill highly proliferative cells, such as *Apc* mutant cells (Shreeram et al., 2002; Blow and Gillespie, 2008).

In summary, we demonstrate that the dynamics of the DNA replication licensing system provides a new way for measuring the proliferative fate of intestinal stem cells. We suggest a model for “working” intestinal stem cells that spend a significant proportion of the G_1_ phase in an unlicensed state until a proliferative-fate decision is made. Correspondingly, exit from the cell cycle in +4 LRCs leads to loss of proliferative capacity and loss of Mcm2 expression causing cells to enter a deeply G_0_ quiescent state (Fig. 6). The unlicensed G_1_ state is lost in *Apc* mutant epithelia, which lack a functional Rb-restriction point. We suggest that the unlicensed G_1_ state serves stem cells in controlling their numbers by regulating the cell cycle.

## Materials and methods

### Mice

All experiments were performed under UK Home Office guidelines. CL57BL/6 (WT), *R26-rtTA Col1A1-H2B-GFP* (H2B–GFP), Lgr5-EGFP-IRES-creERT2 (Lgr5^GFP/+^), and Apc^Min/+^ mice were sacrificed by cervical dislocation or CO_2_ asphyxiation. Fucci2aR mice (Mort et al., 2014) were a gift from R. Mort (University of Edinburgh, Edinburgh, Scotland, UK).

### Tissue preparation: whole small intestine

Dissected pieces of adult-mouse small intestine were washed briefly in PBS and then fixed in 4% PFA for 3 h at 4°C. Intestines were cut into 2 × 2-cm^2^ pieces and fixed overnight in 4% PFA at 4°C. Tissue was embedded in 3% low–melting-temperature agarose and cut into 200-µm sections with a Vibratome (Leica Biosystems). Sections were washed in PBS, permeabilized with 2% Triton X-100 for 2 h, and incubated with blocking buffer (1% BSA, 3% normal goat serum, and 0.2% Triton X-100 in PBS) for 2 h at 4°C. Tissue was incubated in working buffer (0.1% BSA, 0.3% normal goat serum, and 0.2% Triton X-100 in PBS) containing primary antibody Mcm2 (1:500; Cell Signaling Technology) for 48 h at 4°C. Sections were washed five times with working buffer before 48-h incubation with secondary antibodies diluted in working buffer: Alexa Fluor–conjugated goat anti–rabbit (1:500; Molecular Probes) plus 5 µg/ml Hoechst 33342 and Alexa Fluor–conjugated phalloidin (1:150; Molecular Probes). Sections were mounted on coverslips in ProLong Gold (Thermo Fisher Scientific) between 2 × 120-µm spacers.

### Tissue preparation

#### Isolating and staining crypts

Small intestines were dissected, washed in PBS and opened longitudinally. Villi were removed by repeated (≤10 times) scraping of the luminal surface with a coverslip. Tissue was washed in PBS and incubated in 30 mM EDTA (25 min at 4°C), and crypts were isolated by vigorous shaking in PBS. Crypt suspensions were centrifuged (fixed rotor, 88 relative centrifugal force, 4°C), and the pellet was washed twice in cold PBS. Crypts were fixed in 4% PFA (30 min at room temperature), permeabilized in 1% Triton X-100 (1 h at room temperature), and blocked in blocking buffer (2 h at 4°C). Crypts were incubated with primary antibodies diluted in working buffer: Mcm2 (1:500; Cell Signaling Technology), phospo-Histone H3 (1:500; Abcam), Ki67 (1:250, ab15580; Abcam), and αGFP (1:500; Abcam); washed five times with working buffer, before overnight incubation with secondary antibodies diluted in working buffer: Alexa Fluor–conjugated goat anti–mouse or anti–rabbit (1:500; Molecular Probes); or stains: rhodamine-labeled UEA I (1:500), 5 µg/ml Hoechst 33342, or Alexa Fluor–conjugated phalloidin (1:150) at 4°C. Crypts were mounted directly on slides in ProLong Gold overnight.

#### Cytoskeleton buffer extraction of isolated crypts

Soluble proteins were extracted from crypts isolated as described above by incubation with cytoskeleton extraction buffer (10 mM Hepes, 100 mM NaCl, 3 mM MgCl_2_, 1 mM EGTA, 300 mM sucrose, 0.2% Triton X-100, 1 mM DTT, and 2% BSA) supplemented with protease inhibitors (PMSF, pepstatin, leupeptin, cystatin, Na_3_VO_4_, NaF, and aprotinin) for 20 min on ice before fixation. Crypts were then fixed with 4% PFA and processed for imaging as described above.

### H2B–GFP label retention

H2B–GFP expression in transgenic *R26-rtTA Col1A1-H2B-GFP* mice was induced by replacing normal drinking water with 5% sucrose water supplemented with 2 mg/ml doxycycline. After 7 d, doxycycline water was replaced with normal drinking water. Subsequently, mice were sacrificed after 7 d.

### EdU incorporation and detection

Mice were injected i.p. with 100 µg EdU (Thermo Fisher Scientific) prepared in 200-µl sterile PBS. Mice were sacrificed 1 h or 17 h after induction. For organoids, 10 µM EdU was included in crypt medium for 1 h before harvesting. EdU was detected by Click-it chemistry (Thermo Fisher Scientific) by incubation in EdU working buffer (1.875 µM Alexa Fluor 488 azide; Thermo Fisher Scientific), 2 mM CuSO_4_, and 10 mM ascorbic acid overnight at 4°C before processing for immunofluorescence staining.

### Organoid culture

Isolated crypts were dissociated to single cells with TripLE express (Thermo Fisher Scientific) at 37°C for 5 min. Dissociated cells were filtered through a 40-µm cell strainer (Greiner Bio-One) and suspended in growth factor–reduced Matrigel (BD Biosciences). Organoids were grown in crypt medium (advanced DMEM/F12 [ADF] supplemented with 10 mM Hepes, 2 mM Glutamax, 1 mM *N*-acetylcysteine, N2 [Gemini Bio Products], B27 [Thermo Fisher Scientific], and penicillin/streptomycin [MilliporeSigma]) supplemented with EGF/Noggin/R-spondin 1 (ENR) medium (50 ng/ml EGF [Thermo Fisher Scientific], 100 ng/ml Noggin [Thermo Fisher Scientific], and R-spondin–conditioned media) produced from stably transfected L cells (1:4). Chiron99021 (3 µM), valproic acid (1 mM; Thermo Fisher Scientific), and Y27632 (10 µM; Thermo Fisher Scientific) were added to the culture for the first 48 h. Organoids were passaged every 3–5 d by mechanically disrupting Matrigel and by washing and pipetting in ADF. Dissociated crypts were resuspended in fresh Matrigel and grown in crypt medium supplemented with EGFs.

For small-molecule treatments, primary intestinal epithelial cells were cultured in ENR plus Chiron99021, valproic acid, and Y27632 for 3 d, and then, organoids were subcultured in ENR for an additional 2 d before the start of the experiment. Organoids were then treated with the stated small molecules for the indicated times. For induction of unlicensed G_1_, organoids were treated with gefitinib (5 µM), coupled with removal of EGF from the crypt medium. For the reactivation/chase period, the medium was removed and fresh EGFs were added. All EGFs and inhibitors were replenished every 2 d throughout the experiment.

### Flow cytometry and cell sorting

Intestinal crypts were isolated and dissociated to single cells as described above. Isolated cells were filtered through 40-µm cell strainers (Greiner Bio-One). After one PBS wash, organoids were dissociated to single cells by incubation in TrypLE Express (Thermo Fisher Scientific) for 15 min at room temperature, followed by manual disruption by pipetting. Cells were then extracted with cytoskeleton buffer for 20 min on ice, followed by fixation in 0.5% PFA (pH 7.40 for 15 min at room temperature). Cells were then washed once in 1% BSA and permeabilized with ice-cold 70% EtOH for 10 min. Cells were then washed in 1% BSA and resuspended with primary antibodies (Mcm2, 1:500; GFP, 1:500; and Ki67, 1:200) diluted in working buffer overnight at 4°C). After two washes in working buffer, cells were resuspended in secondary antibodies goat anti–mouse or anti–rabbit (Alexa Fluor 647 [1:500; Molecular Probes] or Alexa Fluor 488–Ki67 [1:400, clone SolA15; BD Biosciences]), diluted in working buffer (1 h at room temperature). After two washes in 1% BSA, cells were suspended in working buffer containing 15 µg/ml DAPI. Samples were analyzed on an FACS Canto (BD Biosciences).

For cell sorting, cells were isolated from Lgr5–GFP mice as described above by treatment with TrypLE Express for 15 min at 37°C, followed by filtration through 40-µm filters (Greiner Bio-One). Cells were sorted in ADF supplemented with 1% FBS and DAPI (15 µg/ml). Sorting was performed with an Influx cell sorter (BD Biosciences). Cells were checked after being sorted to ensure sample purity by reexamining Lgr5 expression in the sorted gates.

### Microscopy and image analysis

Samples were imaged with an LSM 710 microscope (Carl Zeiss) with 25×/0.8 NA and 40×/1.3 NA LD Plan-Neofluar objective lenses and immersion oil with a refractive index of 1.516. Z stacks were acquired at optimal-section intervals between 0.3 and 0.8 µm at room temperature with Zen 2011 acquisition software (Carl Zeiss). All fixed samples were mounted in ProLong Gold. For quantification, images were acquired at 16 bit-depth.

Image processing and analysis were performed with Imaris software (Bitplane). Images of individual crypts were manually cropped, ensuring that an individual crypt was the only region of interest. All nuclei were detected in individual crypts with automated thresholding in Imaris, with the measurement-point function set to detect nuclei at an estimated size of 3.5 µm. Missed or incorrectly assigned nuclei were manually identified. This function produced measurement points that segmented the specific region at the corresponding coordinate of the measurement point. Mean intensities for different channels were calculated per spot. This equates to the intensity at the center region of each nucleus. A reference nucleus at the crypt base was used to define the crypt-base position. The Euclidean distance to that point was measured and defined as the distance to the crypt base. Multiple images were analyzed with the same workflow, and the analyzed files were collated. For vibratome sections, a plane was manually defined running through to the muscle layer beneath the epithelium. The smallest distance to that surface was defined for segmented nuclei. For nuclear-volume estimation, the nuclei were manually segmented in 3D with the manual segmentation tools within Imaris (Fig. S1).

### Flow cytometry analysis

Flow cytometry data were analyzed with FlowJo software (Tree Star) and a standardized gating strategy (Fig. S1). In brief, cells were identified by forward and side light scatter. After doublet discrimination, gates were set with appropriate controls lacking conjugated secondary antibodies and without primary antibodies. Mcm2-negative gates were set by secondary-only controls in conjunction with the Mcm2 intensity of G_2_ cells. G_1_ cells were discriminated based on the maximal DNA-bound Mcm2 intensity before the S phase and by DAPI intensity.

### Computational modeling

A deterministic computer model for licensing in G_1_ was written in the Swift programming language (Apple) in the Xcode 9 development environment. The model assumes that there is a minimum G_1_ period that is required for cells to grow to a critical size before they can enter the S phase. Licensing can take place during that period at a constant rate. A licensing rate of 1 means that cells will be fully licensed in exactly the minimum G_1_ period. It was also assumed that cells had a robust “licensing checkpoint” (Shreeram et al., 2002; Blow and Gillespie, 2008), so that they could not enter the S phase until the origins have been fully licensed. An optional “unlicensed G_1_ period” occurred at the start of G_1_; during which time, no licensing takes place. In the simulation, cells enter G_1_, wait for any optional “unlicensed G_1_ period,” then start to license origins at a fixed rate; cells exit G_1_ and modeling ceases only when they have become maximally licensed and the minimum G_1_ period had elapsed. The model divided the minimum G_1_ period into 10,000 equal steps and recorded the degree of licensing at the end of each step in the licensing array. The contents of the licensing array were distributed into 101 different frequency bins, ranging from 0 (no licensing) to 100% (maximal licensing). To model the background signal recorded by flow cytometry, all the licensing values were increased by 5%. To model the flow cytometry measurement error, the frequency array was smoothed by starting at the smallest bin and pushing 80% of the cell counts into the next largest bin and, then, starting at the largest bin, pushing 80% of the cell counts into the next-smallest bin.

### Online supplemental material

Fig. S1 illustrates the image-analysis workflow used to measure licensing dynamics along the crypt axis and describes the flow cytometry gating strategy. Fig. S2 shows the clonal cell-cycle field effects reflected by neighboring cells along the crypt axis with similar licensing and replication profiles, and the flow cytometry and nuclear volume measurements are used to show that intestinal stem cells have a normal cell-cycle distribution and are not all in the G_2_ phase of the cell cycle. Fig. S3 shows the ubiquitous expression of Ki67 along the crypt axis. Fig. S4 examines the expression of the fluorescence ubiquitination cell-cycle indicator and reporters in the intestinal epithelium, showing that most stem cells express little of the G_1_ hCdt1(30/120) marker, consistent with an unlicensed G_1_ phase. Fig. S5 shows the licensing dynamics in the intestinal organoids with altered stem cell–niche signaling. This revealed that (a) the combination of Ki67 and DNA-bound Mcm2 is a robust means of exploring cell-cycle dynamics during transitions to quiescence, (b) Chir99021 and valproic acid treatments induce unique cell-cycle dynamics, and (c) Apc^Min/Min^ organoids are sensitive to gefitinib, whereas WT organoids are not. Video 1 shows cell-cycle clones. We also include the source code used for the simulations of licensing through G_1_.

## Supplementary Material

Supplemental Materials (PDF)

Source code (PDF)

Video 1
