# Supplementary material for: Lgr5+ intestinal stem cells reside in an unlicensed G1 phase
Source: J Cell Biol. 2018 May 7;217(5):1667–85. doi: 10.1083/jcb.201708023 (PMC5940300; doi:10.1083/jcb.201708023)
Supplement: Supplemental Materials (PDF) [file JCB_201708023_sm.pdf]

JCB

Supplemental material

Carroll et al., https://doi.org/10.1083/jcb.201708023

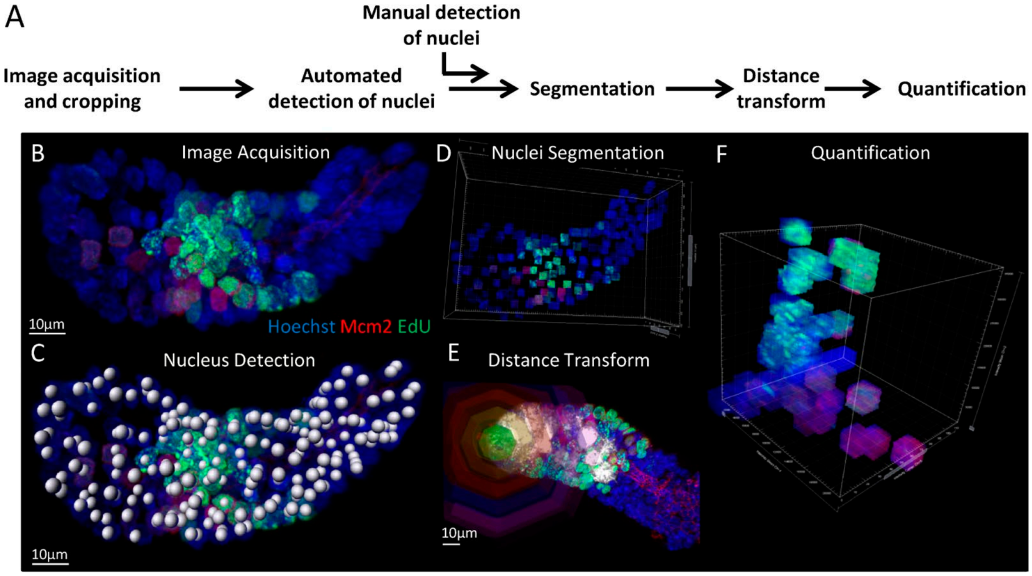

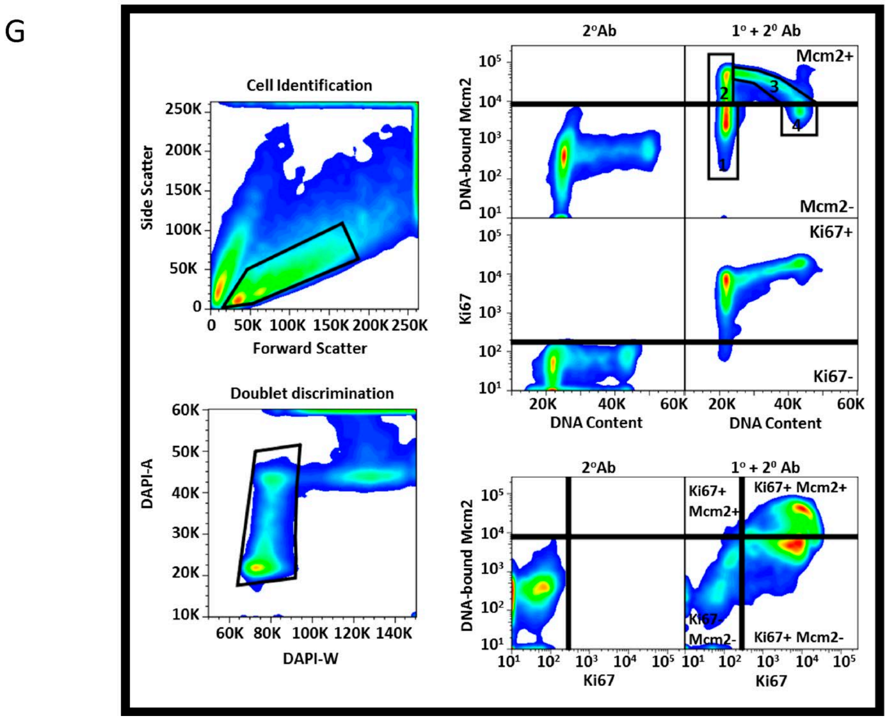

Figure S1. **Image analysis. (A)** Image analysis workflow. **(B)** Representative image of an extracted isolated crypt 1 h after an EdU pulse (green) stained with Hoechst (blue) and an antibody against Mcm2 (red). Bar, 10 µm. **(C)** Detection of nuclei in the crypt in A in 3D. Nuclei were detected in 3D with the segmentation tools in Imaris. Detection was validated visually or in each individual crypt. Bar, 10 µm. **(D)** Segmentation of the region of interest defined by nuclei detection in B. **(E)** A distance transform was performed in Imaris to measure the distance of each nucleus to a reference nucleus at the crypt base. Visual representations of distances divided into different bins are displayed (green, 0–10 µm; yellow, 10–20 µm; red, 20–30 µm; blue, 30–40 µm; and magenta, 40–50 µm). Bar, 10 µm. **(F)** Representative 3D quantification of the crypt in A shows the distance from the crypt base (x axis), DNA-bound Mcm2 (y axis), and EdU incorporation (z axis). **(G)** An overview of the gating strategy used for flow cytometry experiments. Intestinal epithelial cells were separated from debris based on forward and side light scatter. Single cells were subsequently distinguished from doublets based on pulse-width gating. Negative gates were set on control samples stained with only secondary antibody. Unlicensed G_1 (population 1) and licensed G_1 (population 2) were distinguished based on DNA content and based on the threshold set by the negative control samples. S phase cells (population 3) are shown as an arc of cells emanating from the fully licensed cell population, increasing in DNA content and losing DNA-bound Mcm2. G_2/M cells (population 4) is shown as a population with 4N DNA content and lacking DNA-bound Mcm2. Data are representative of 24 experiments.

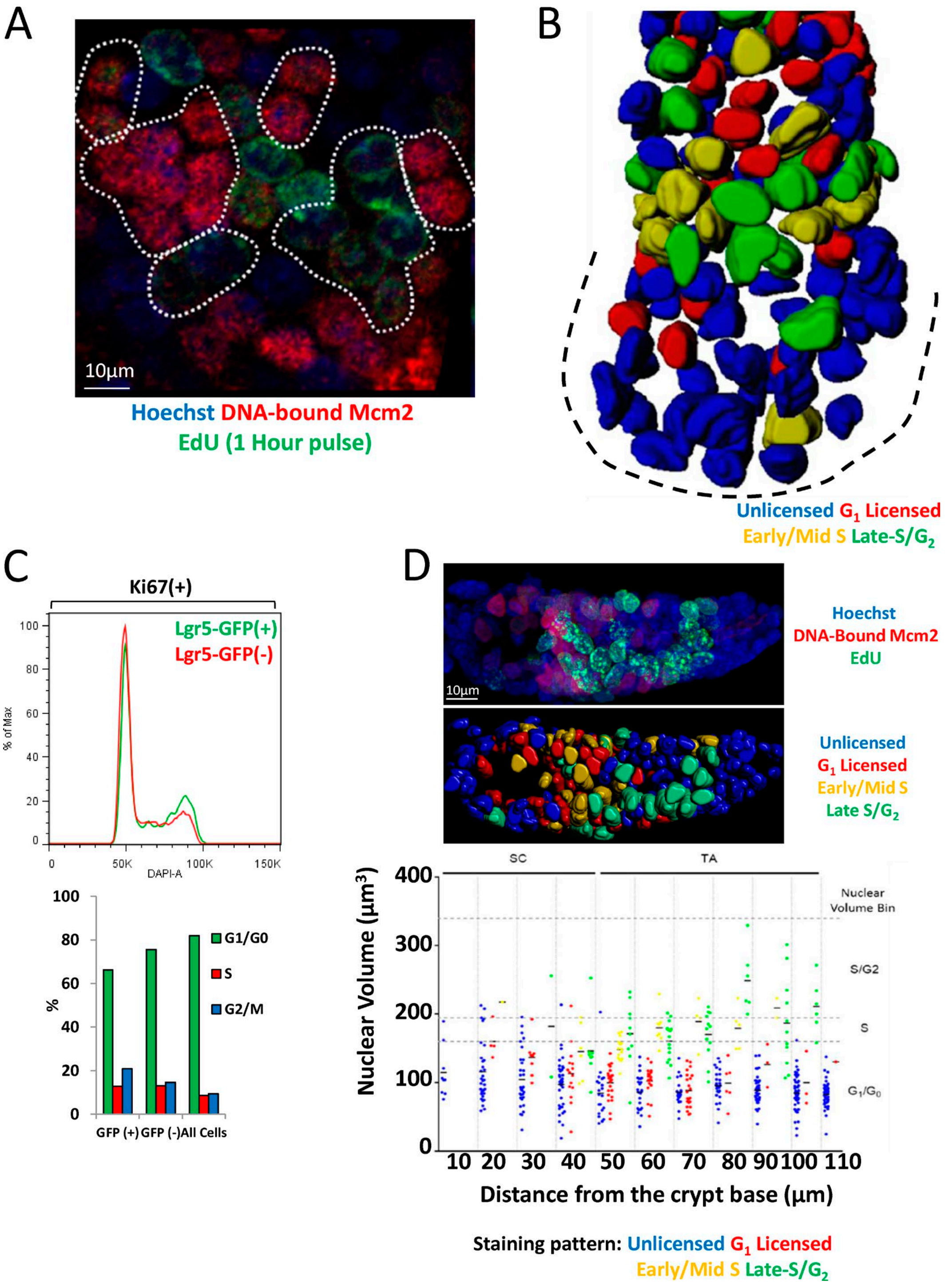

Figure S2. **Clonal cell-cycle patterns in the intestinal epithelium. (A)** Representative section through an extracted crypt after a 1-h EdU pulse (green) and stained with Hoechst (blue) and an antibody against Mcm2 (red). Discrimination of cell-cycle staging using DNA-bound Mcm2 and EdU incorporation patterns allows visualization of clonal cell-cycle field effects revealing many neighboring cells with similar DNA-bound Mcm2 and DNA replication patterns. These clones may represent lineages of from single cells that progress through the cell cycle at the same rate. Bar, 10 µm. **(B)** Representative image of an isolated crypt in which a surface rendering was performed on all nuclei, and color codes were applied to reflect cell-cycle stage. Representative cell-cycle distributions for isolated Ki67$^+$, Lgr5$^+$, and Lgr5$^-$ intestinal epithelial cells are shown. **(C)** Representative cell-cycle distributions for isolated Ki67$^+$, Lgr5$^+$, and Lgr5$^-$ intestinal epithelial cells. The mean of each cell-cycle phase is displayed (n = 2). **(D)** Nuclear volumes were rendered for individual nuclei in whole intestinal crypts isolated 1 h after labeling with EdU. Image shows nuclei (blue), EdU (green), and licensed Mcm2 (red). Maximum intensity projections of the original image are displayed at the top and corresponding rendered nuclei at the bottom. Bar, 10 µm. Nuclear surfaces were color-coded according to cell-cycle states: blue, unlicensed; red, licensed G_1; yellow, S phase; and green, late S/G_2. Nuclear volumes were measured for all nuclei in representative crypts (n = 3). Unlicensed (n = 368); G_1 (n = 104); S phase (n = 41); and late S/G_2 (n = 70) and the distance of cells from the crypt base were binned into 10-µm intervals. Known parameters of the nuclear volume for known cell-cycle stages (Fig. 3 B) are overlaid.

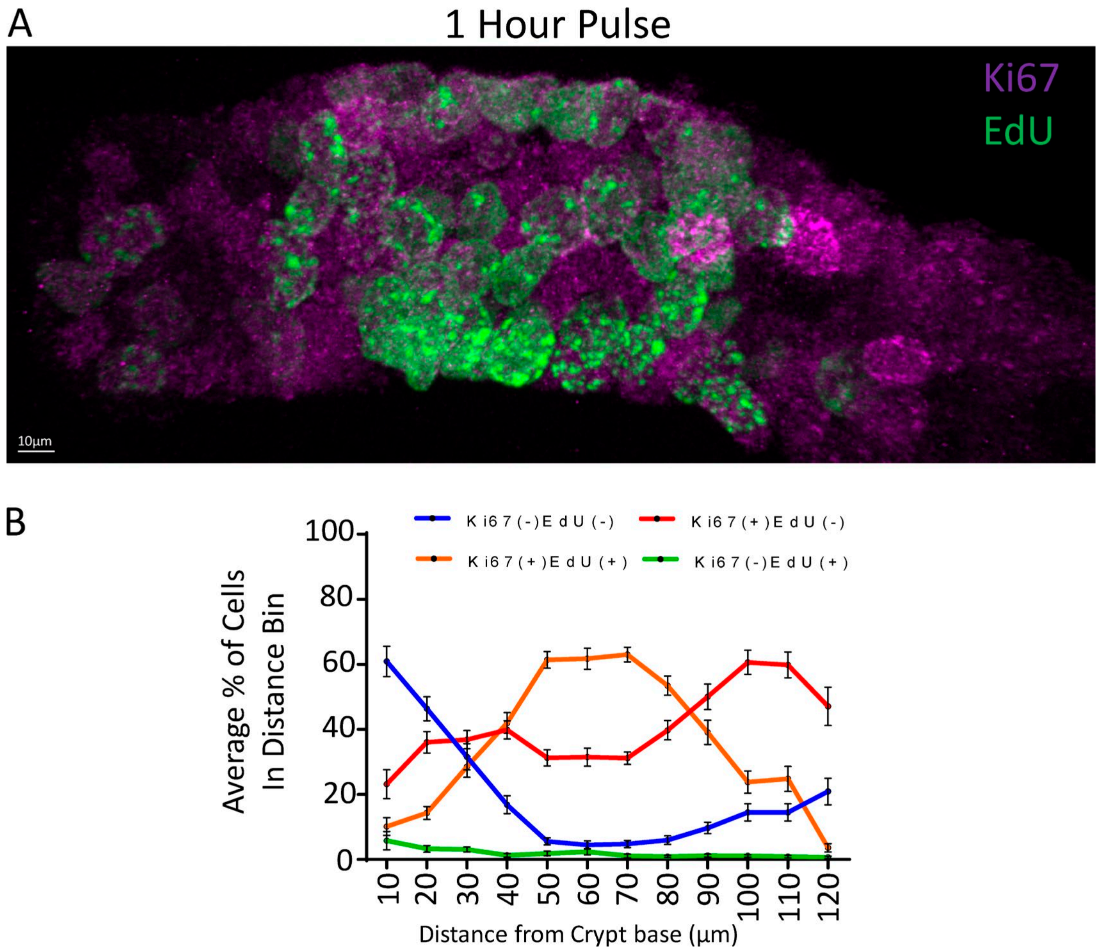

Figure S3. **Ki67 expression along the crypt–villus axis. (A)** A representative, isolated crypt 1 h after labeling with EdU (green) and stained with an antibody against Ki67 (magenta). Bar, 10 µm. **(B)** Quantification of the distribution of $Ki67^+$ cells along the crypt axis. Cells were binned into four groups: (1) $Ki67^-$ and EdU^-; (2) $Ki67^+$ EdU^-; (3) $Ki67^+$ EdU^+; (4) $Ki67^-$ EdU^+; and by their distance from the crypt base. Data are displayed as the percentage means of a particular cell subtype per distance bin and as means ± SEM. Data from 75 crypts are displayed (*n* = 14,264 cells).

https://doi.org/10.1083/jcb.201708023

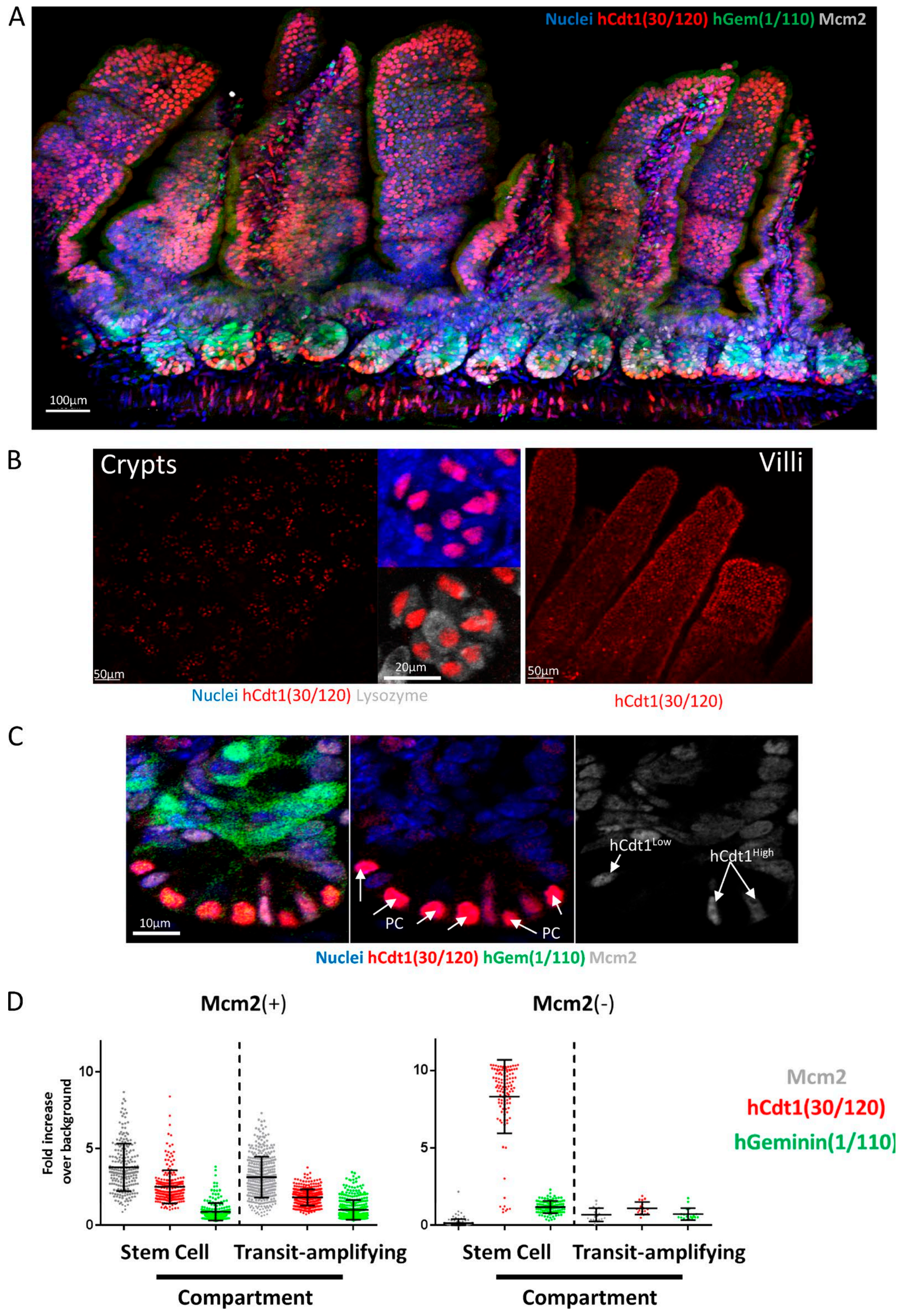

Figure S4. **Delayed accumulation of Fucci2aR reporter hCdt1(30–120) in intestinal stem cells. (A)** Representative image of a vibratome section of intestinal tissue derived from Fucci2aR mice, stained with Hoechst (nuclei) and an antibody against Mcm2, and showing expression of the G_1 marker hCdt1(30–120) and S/G_2/M marker hGeminin(1/110), Bar, 100 µm. **(B)** Representative image hCdt1(30–120) expression in the crypt base (left; Bars: [left] 50µm; [right panels] 20 µm) and villi (right; Bar, 50 µm). Most cells in the crypt base that highly express hCdt1(30–120) are UEA$^+$ Paneth cells. **(C)** A representative image of a Fucci2aR intestinal crypt stained with Hoechst and with an antibody against Mcm2. All Mcm2$^-$ Paneth cells in the crypt base express high levels of hCdt1(30–120). Most Mcm2$^+$ stem cells in the crypt base express very low levels of hCdt1(30–120) (hCdt1^{low}). Only a few Mcm2$^+$ cells were found to express high levels of hCdt1(30–120) (hCdt1^{high}). Bars, 10 µm. Many cells in the TA compartment expressed hGeminin(1/110). **(D)** Quantification of the expression of Mcm2, hCdt1(30/120), and hGeminin(1/110) in Mcm2$^+$ and Mcm2$^-$ cells in the stem cell and TA compartments. Data are displayed as a ratio of signal to the background signal detected in negative cells. Stem cell compartment: Mcm2$^+$, n = 220, and Mcm2$^-$, n = 112; and TA compartment, Mcm2$^+$, n = 383, and Mcm2$^-$, n = 18.

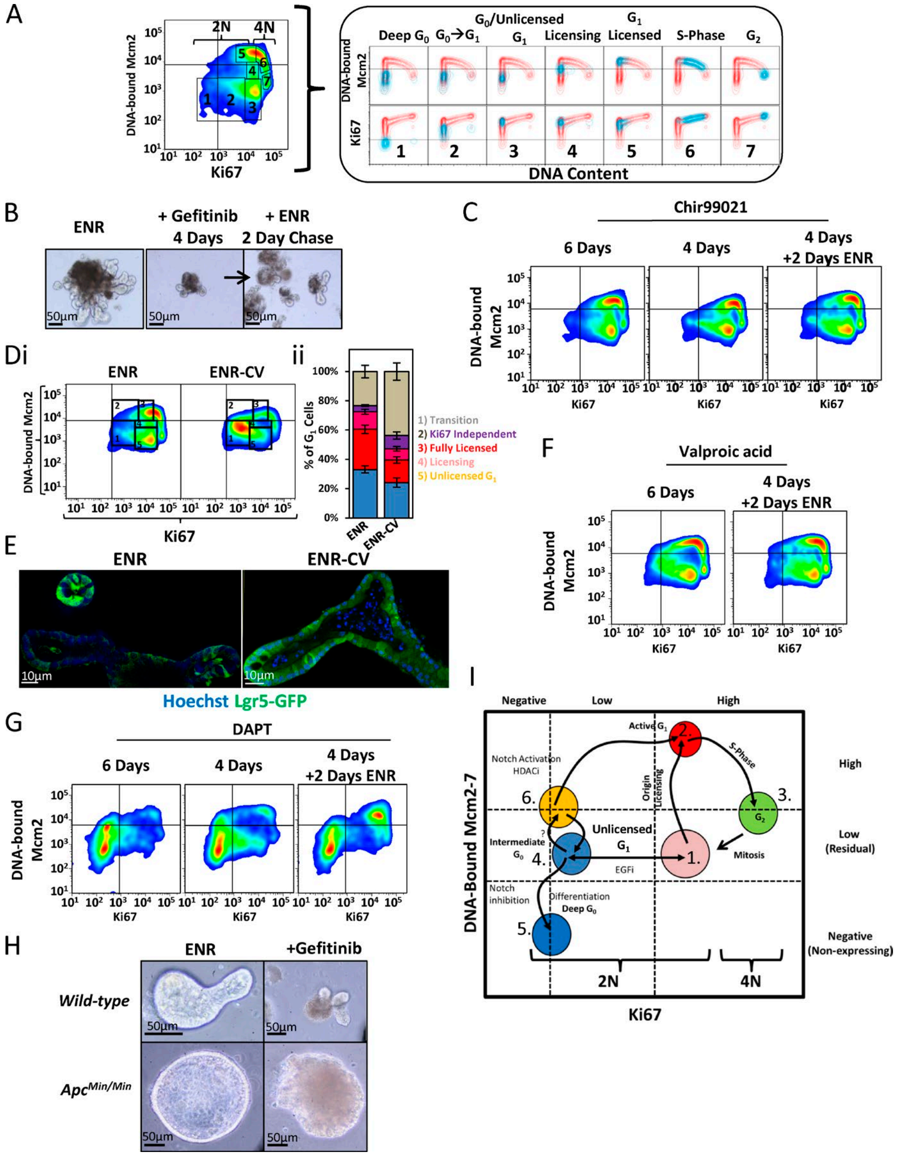

Figure S5. **Manipulation of the stem cell niche can artificially induce unlicensed G_1. (A)** Representative flow cytometry profile of extracted epithelial cells isolated from organoids in ENR medium. The displayed image is the same profile displayed in Fig. 5 C. The populations in boxed regions 1–7 are overlaid onto the individual Mcm2 and Ki67 cell-cycle profiles plotted against DNA content (right). **(B)** Representative bright-field images of organoids in normal (ENR) medium, treated with gefitinib for 4 d, and after 2 d in fresh ENR medium after Gefitinib treatment. Bars, 50 µm. **(C)** Representative flow cytometry profiles from extracted cells isolated from organoids treated with 10 µM Chir99021 for the indicated time and after 2 d in fresh ENR medium after Chir99021 treatment. Data are representative of three independent experiments. **(D)** Representative flow cytometry profiles from extracted cells isolated from organoids treated with normal medium (ENR) or in normal medium containing Chir99021 and valproic acid (ENR-CV). Displayed is the comparison of DNA-bound Mcm2 versus Ki67 content (i). The gated populations (1) transition (unlicensed $G_1 \leftrightarrow$ intermediate G_0), (2) Ki67 independent origin licensing pathway, (3) fully licensed G_1 cells, (4) normal licensing, (5) unlicensed G_1 were quantified (n = 3). **(E)** Representative images of Lgr5–GFP organoids treated with ENR or ENR-CV. Bars, 10 µm. In ENR-CV–treated organoids, most cells express Lgr5. **(F)** Representative flow cytometry profiles from extracted cells isolated from organoids treated with valproic acid for the indicated times and after 2 d in fresh ENR medium after valproic acid treatment. Data are representative of two independent experiments. **(G)** Representative flow cytometry profiles from extracted cells isolated from organoids treated with DAPT for indicated times and after 2 d in fresh ENR medium after DAPT treatment. Data are representative of two independent experiments. **(H)** Representative images of WT and *Apc*$^{Min/Min}$ intestinal organoids after treatment with 5 µM gefitinib for 3 d. Data are representative of two independent experiments. Bars, 50 µm. **(I)** Model for the unique cell-cycle characteristics of organoid epithelial cells. Normal, highly proliferative cells express Ki67 and Mcm2 proteins that are not DNA bound (1). During a normal cell cycle, cells are activated from unlicensed G_1 and rapidly license origins (2). Mcm proteins are subsequently displaced during DNA replication and remain unlicensed through G_2 (3). Inhibiting EGFR causes highly proliferative cells (Ki67^{hi}) to arrest in unlicensed G_1 with maintained Mcm2 protein expression. Prolonged EGFR inhibitor (EGFi) treatment causes transition into an intermediate state of G_0 accompanied by loss of Ki67 expression (Ki67^{lo}), but maintenance of MCM2–7 protein expression (4). Induction of terminal differentiation by inhibition of Notch signaling is associated with a terminal loss of MCM2–7 proteins and entry into deep G_0 (5). Notch activation (or histone deacetylase inhibition [HDACi]) induces a unique subset of Ki67^{lo} unlicensed G_1 cells to license origins independent of Ki67 (6). We suggest that the unique cell population observed upon ENR and ENR-CV/ENR-V (no Chir99021) treatment may be a reserve subset of stem cells that express Lgr5 and start expressing MCM2–7 and enter unlicensed G_1 from deep G_0. These cells have unique cell-cycle characteristics and can immediately license origins independent of Ki67 expression ($6 \rightarrow 2 \rightarrow 3$).

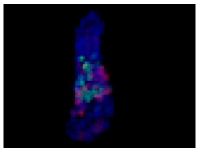

Video 1. **Cell-cycle clones.** A 3D rotation of a representative intestinal crypt showing nuclei (blue), DNA-bound Mcm2 (red), and EdU (green; 1-h pulse). The crypt base is at the bottom of the image. An isosurface rendering of nuclei within the crypt has also been performed and has been colored to match specific cell-cycle stages: unlicensed (blue), G_1 licensed (red), early S phase (yellow), and late S/G_2 (green).

Provided online as a PDF is the source code used for the simulations of licensing through G_1.
