# Supplementary material for: Lgr5+ intestinal stem cells reside in an unlicensed G1 phase
Source: J Cell Biol. 2018 May 7;217(5):1667–85. doi: 10.1083/jcb.201708023 (PMC5940300; doi:10.1083/jcb.201708023)
Supplement: Source code (PDF) [file JCB_201708023_DataS1.pdf]

```
//
//  JBLicensingRateModel.swift version 1.0
//
//  Created by Julian Blow on 05/10/2017.


import Foundation

class JBLicensingRateModel {
    let licensingRate: Double    // rate of licensing in proportion to minimum G1 length
    let proportionUnlicensedG1: Double      // proportion of minimum G1 before licensing occurs
    let timeStepsForMinimumG1: Int
    let licensingPerStep: Double
    let timeStepsBeforeLicensingStarts: Int
    let binsForFACS: Int
    let backgroundForFACS: Double
    let binSmoothingProportion: Double
    let totalNumberOfCells: Double
    let precisionCutOffForSmoothing: Double
    let numberOfTimepointsOutput: Int

    init (licensingRate: Double, proportionUnlicensedG1: Double) {
        self.licensingRate = licensingRate
        self.proportionUnlicensedG1 = proportionUnlicensedG1
        timeStepsForMinimumG1 = 10000
        licensingPerStep = (licensingRate / Double(timeStepsForMinimumG1)) * 100
        timeStepsBeforeLicensingStarts = Int(proportionUnlicensedG1 * Double(timeStepsForMinimumG1))
        binsForFACS = 100
        backgroundForFACS = 5
        binSmoothingProportion = 0.8
        totalNumberOfCells = 10000.0
        precisionCutOffForSmoothing = totalNumberOfCells / 10000      // cutoff for the smallest number of cells we continue to
smooth for the far right of the array
        numberOfTimepointsOutput = 100
    }

    func run () -> ([Double], [Double])  {
        var licensingTimepoints: [Double]
        if licensingRate > 0 { licensingTimepoints = simulateLicensing() }
        else { licensingTimepoints = Array(repeating: 0.0, count: timeStepsForMinimumG1) }       // if licensing can never occur
        let FACSOutputExact = simulateFACSExact(licensingTimepoints: licensingTimepoints)
        let FACSOutput = smoothFACSOutput(FACSOutputExact: FACSOutputExact)
        var simplifiedLicensingTimepoints = [Double]()
        for index in stride(from: 0, to: licensingTimepoints.count, by: licensingTimepoints.count/numberOfTimepointsOutput) {
            simplifiedLicensingTimepoints.append(licensingTimepoints[index])
        }
        return (FACSOutput, simplifiedLicensingTimepoints)
    }

    func simulateLicensing() -> [Double] {
    // simulates the degree of MCM loading given the licensingRate and proportionUnlicensed
    // returns amount of MCM loaded (percent max) at different time points
        var amountOfLicensing = 0.0
        var licensingArray = Array(repeating: 0.0, count: timeStepsBeforeLicensingStarts)
        var timeStep = timeStepsBeforeLicensingStarts

        repeat {
            amountOfLicensing += licensingPerStep
            if amountOfLicensing > 100 { amountOfLicensing = 100 }
            licensingArray.append(amountOfLicensing)
            timeStep += 1
        } while (amountOfLicensing < 100) || (timeStep < timeStepsForMinimumG1)

        return licensingArray
    }

    func simulateFACSExact (licensingTimepoints: [Double]) -> [Double] {
    // takes an array amount of MCM loaded at different time points as created by simulateLicensing()
    // turns this into a simulated output from a FACS (flow cytometry) analysis with totalNumberOfCells
        var maxMCMValue = licensingTimepoints.max()! // can be >100 because of smoothing
        if maxMCMValue == 0 { maxMCMValue = 100 }      // if no licensing occurred
        var FACSOutputExact = Array(repeating: 0.0, count: binsForFACS+1)        // binsForFACS+1 because we 0 and 100 are
both possible
        let numberOfCellsPerTimepoint = totalNumberOfCells / Double(licensingTimepoints.count)        // so we get
totalNumberOfCells events
```

```
        for anMCMContent in licensingTimepoints {
            let binIndex = Int(anMCMContent * Double(binsForFACS) / maxMCMValue)
            assert(binIndex <= binsForFACS, "Cannot have an MCM content greater than 100, the bin number")
            if binIndex>=0 { FACSOutputExact[binIndex] += numberOfCellsPerTimepoint }            // add cells to bin
        }

        return FACSOutputExact
    }

    func smoothFACSOutput (FACSOutputExact: [Double]) -> [Double] {
        // simulates the error in FACS data by:
        // first adding a background of backgroundForFACS to all samples
        // then pushing binSmoothingProportion of each bin into the higher bin, when the value > cutOffForDither
        // then pushing binSmoothingProportion of each bin into the lower bin
        let backgroundBins = Array(repeating: 0.0, count: Int(backgroundForFACS))
        var FACSOutput = backgroundBins + FACSOutputExact       // add the background of empty pbins
        var binIndex = 0
        var amountToShift = 0.0
       repeat {                        // expand rightwards by dithering
            amountToShift = FACSOutput[binIndex] * binSmoothingProportion
            FACSOutput[binIndex] -= amountToShift
            if binIndex+1 < FACSOutput.count { FACSOutput[binIndex+1] += amountToShift }
            else { FACSOutput.append(amountToShift) }
            binIndex += 1
            } while (amountToShift >= precisionCutOffForSmoothing) || (binIndex < FACSOutputExact.count + Int(backgroundForFACS))

       binIndex = FACSOutput.count-1
        while binIndex >= 0 {                  // expand leftwards by dithering
            amountToShift = FACSOutput[binIndex] * binSmoothingProportion
            FACSOutput[binIndex] -= amountToShift
            if binIndex > 0 { FACSOutput[binIndex-1] += amountToShift }
            binIndex -= 1
        }

        return FACSOutput
    }
```
